# An Efficient, One-Pot Transamidation of 8-Aminoquinoline Amides Activated by Tertiary-Butyloxycarbonyl

**DOI:** 10.3390/molecules24071234

**Published:** 2019-03-29

**Authors:** Wengang Wu, Jun Yi, Huipeng Xu, Shuangjun Li, Rongxin Yuan

**Affiliations:** 1 College of Chemistry, Chemical Engineering and Materials Science of Soochow University, 199 Ren’ai Road, Suzhou, Jiangsu 215123, China; 20155209006@suda.edu.cn; 2 Jiangsu Laboratory of Advanced Functional Material, School of Chemistry and Materials Engineering, Changshu Institute of Technology, Changshu 215500, China; xuhp150315217cslg@126.com (H.X.); lisj150315206cslg@126.com (S.L.)

**Keywords:** one-pot reaction, amide C_(acyl)_–N cleavage, palladium, N-heterocyclic carbene, directing group removal

## Abstract

The efficient, one-pot access to the transamidation of 8-aminoquinoline (8-AQ), notorious for its harsh removal conditions, has been widely employed as an auxiliary in C–H functionalization reactions due to its strong directing ability. In this study, the facile and mild Boc protection of the corresponding 8-AQ amide was critical to activate the amide C_(acyl)_–N bond by twisting its geometry to lower the amidic resonance energy. Both aryl and alkyl amines proceeded transamidation in one-pot, user-friendly conditions with excellent yields.

## 1. Introduction

**Scheme 1:**
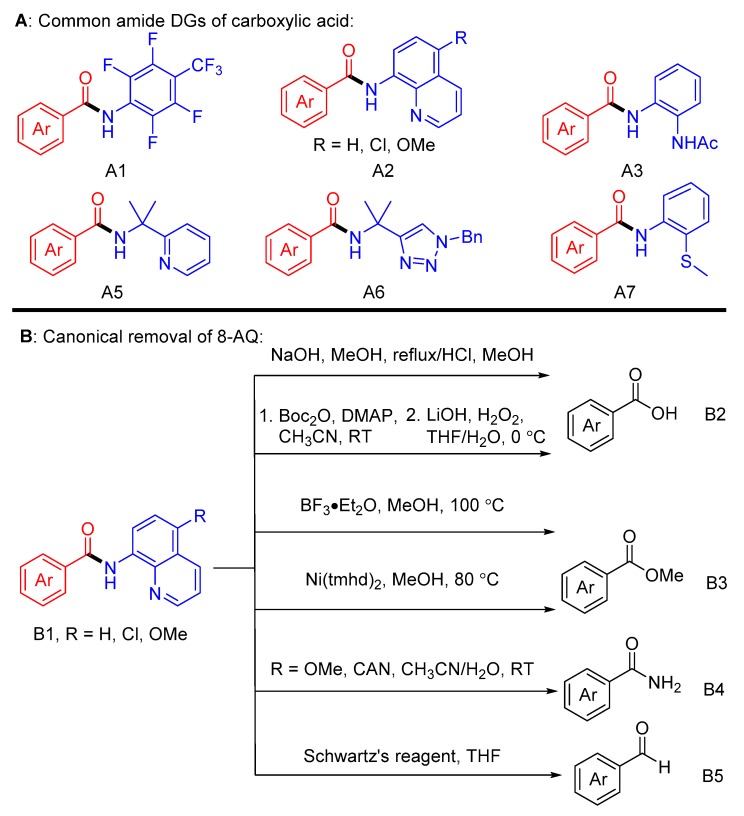
Amide directing groups (DGs) in C–H activation, removal methods, and our design.

C–H functionalization has emerged as a powerful strategy for building-up new molecules [1,2,3,4]. In the past decades, the growing interest in C–H functionalization has led to increased attention to this paradigm for organic synthesis, which has inspired exceptional advances in this field [5,6,7,8]. The maneuver of directing groups (DGs) in C–H functionalization has been proved to be pivotal to facilitate the desired transformation. Common DGs include ketones [9,10,11], esters [12,13,14], carboxylic acid [15,16,17], amides [18,19,20,21,22], etc. Among them, amides are one of the most frequently used motifs (Scheme 1(A)). Taking advantage of its strong coordination with transition metals, a myriad of contributions have been made, especially with bidentate amide DGs. In 2005, Daugulis [23] introduced 8-aminoquinoline (8-AQ) A2 to C–H functionalization, which has been widely used in Pd- [24,25,26,27], Rh- [28,29,30], Ru- [31], Ni- [32,33,34,35,36,37,38], Cu- [39,40,41], Co- [42,43,44], and Fe-catalyzed [45,46,47,48] C–H activation to enable various reactions.

**Figure 1:**
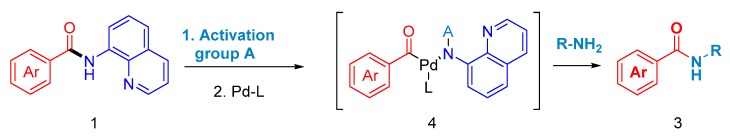
New strategy to remove 8-aminoquinoline (8-AQ).

As a result of electron delocalization (n_N_ to π_C═O_*, ∆H_rot_ of ca. 15–20 kcal/mol in planar amides) [49], the difficulties in uninstalling amide DGs greatly constrain its application (Scheme 1B). Conventional deprotection required stoichiometric amounts of a strong base or acid (Scheme 1(B,B2)), which diminished the functional tolerance [37]. A milder, two-step strategy was therefore widely used, where 8-AQ amides were firstly transformed to tertiary amides by using di-*tert*-butyl decarbonate (Boc_2_O), which was subsequently hydrolyzed by lithium hydroxide and hydrogen peroxide (B2) [50]. Similarly, transesterification strategies by boron trifluoride-diethyl etherate (BF_3_·Et_2_O) were also limited due to the harsh Lewis acid BF_3_ (B3) [37]. Recently, Morimoto and Ohshima reported nickel-catalyzed alcoholysis of unactivated 8-aminoquinoline amides in neutral conditions with excellent functional compatibilities (B3); however, it is mostly limited to alkyl amides [51]. Inspired by the para-methoxyphenyl (PMP) protecting group for amines, Chen installed a methoxy group at the 5 position of 8-AQ for facile removal with ceric ammonium nitrate (CAN) under mild conditions (B4) [52]. Unfortunately, the modified auxiliary is expensive due to its multistep synthesis. Reduction with stoichiometric zirconocene hydrochloride (also known as Schwartz’s reagent) gives the corresponding aldehyde, yet this organometallic reagent is not user friendly [53]. Compared with the booming C–H functionalization reactions, the removal of auxiliary groups is still lagging behind. Meanwhile, Garg [54,55,56,57,58,59,60,61,62,63,64] and Szostak [65,66,67,68,69,70,71,72,73,74,75,76,77,78,79,80,81,82,83] proved that by increasing the sterics of the substitution group, the formation of twisted amides activates the amidyl C_(acyl)_–N bond to favor the insertion of low-valent metals, furnishing the acyl−metal intermediate for further transformations. Inspired by these advances in amidyl C_(acyl)_–N activation, we envisioned that 8-AQ removal could be achieved in this manner after C–H functionalized reactions (Figure 1).

## 2. Results

In order to test our proposal, first, the proper activating group of 8-AQ was chosen. We decided to choose Boc as the activating group for three reasons: (1) The installation of Boc was very moderate, which enhanced the functional group tolerance, with a nearly quantitative yield [84,85,86,87]. (2) 8-aminoquinoline can be easily recycled through deprotection of the *tert*-butyl quinolin-8-ylcarbamate byproduct. (3) Above all, Boc is one of the most thriving activation groups in amidyl C_(acyl)_–N transformation reactions.

Our study was initiated using 3-methyl-*N*-(quinolin-8-yl)benzamide (**1a**) with aniline (**2a**) as model substrates for transamidation in the presence of different bases (Table 1). We started screening with well-developed phosphine ligands. Unfortunately, neither PPh_3_ nor PCy_3_ gave a product. The more σ-donating N-heterocyclic carbenes (NHCs) have revealed high efficiency in inert bond activation, such as C–O [88], C–F [89], as well as amide transformation reactions [63,65,70]. As expected, when using NHC ligands, the desired product was observed. With these promising results, several NHC ligands were examined for transamidation reactions (entries 3–9). Although the combination of Ni(COD)_2_ and SIPr (entry 9) showed a higher yield than (IPr)Pd(cinnamyl)Cl (entry 6), due to the significant difference of stability under air, we decided to use (IPr)Pd(cinnamyl)Cl as the catalyst, which was more user friendly [77]. A higher temperature was critical to improve the yield (entry 10). Neither the strong base sodium *tert*-butoxide (entry 12) nor the organic base 1,8-diazabicyclo[5.4. 0]undec-7-ene (DBU) (entry 13) enhanced the yield. Switching the reaction solvent from THF to MeCN, dioxane or DMF resulted in lower yields (entries 14–16). The control experiment showed that the Pd catalyst was indispensable (entry 17). Finally, we attempted a one-pot transamidation (entry 18) and found that there was no detriment to the yield. The separation of 1a and 4-dimethylaminopyridine (DMAP) was not needed, which enhanced the step efficiency and made the reaction greener.

**Figure 2:**
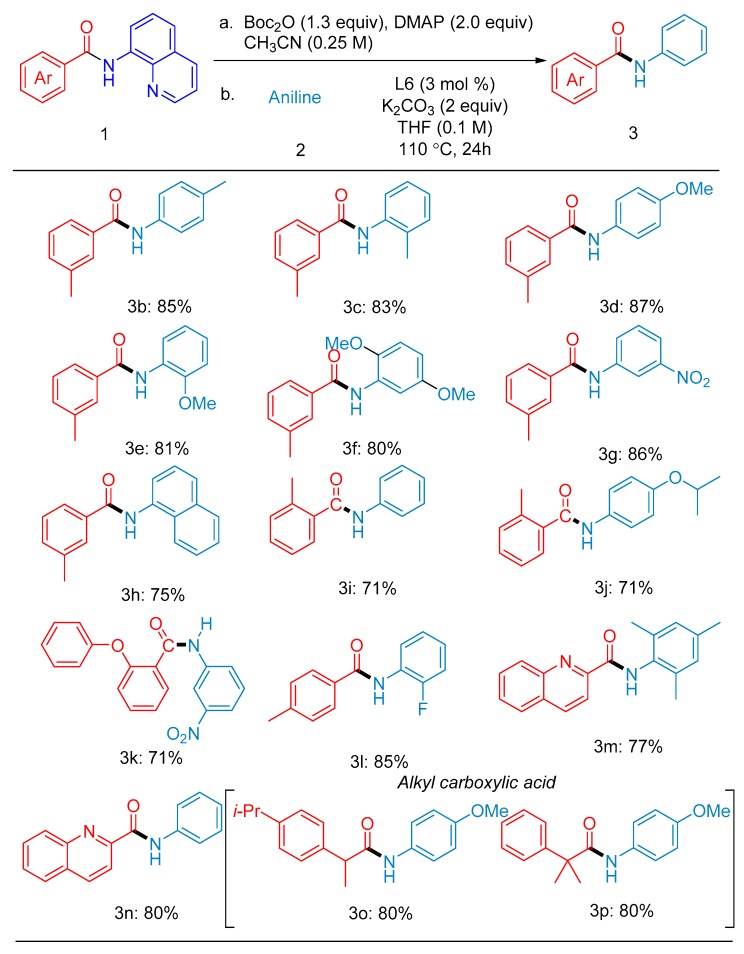
Scope of the transamidation reaction. Conditions: see Supplementary Materials for details.

With the optimized condition in hand, the substrate comparability of the reaction was examined (Figure 2). First, we tested the amine scope. Methyl substitution on both *para* (**3b**) and *ortho* (**3c**) positions gave good yields, although the *ortho* position was more hindered. The electronic effect of the substitution groups did not have a great impact on the yield. Strong electron-donating (**3f**) as well as electron-withdrawing (**3g**) groups were all tolerated. Next, we tested the carboxylic acid part of the amide. The reaction still was not sensitive to the hindrance, as even the bulky *ortho*-phenoxy group gave an excellent yield (**3k**). Heterocycles such as quinoline (**3m**, **3n**) did not corrupt the efficiency of the reaction, while it was noteworthy that even the challenging di-*ortho*-substituted aniline was tolerated (**3m**). More strikingly, aliphatic carboxylic acids, such as ibuprofen-derived species (**3o**), were also workable in this condition.

**Figure 3:**
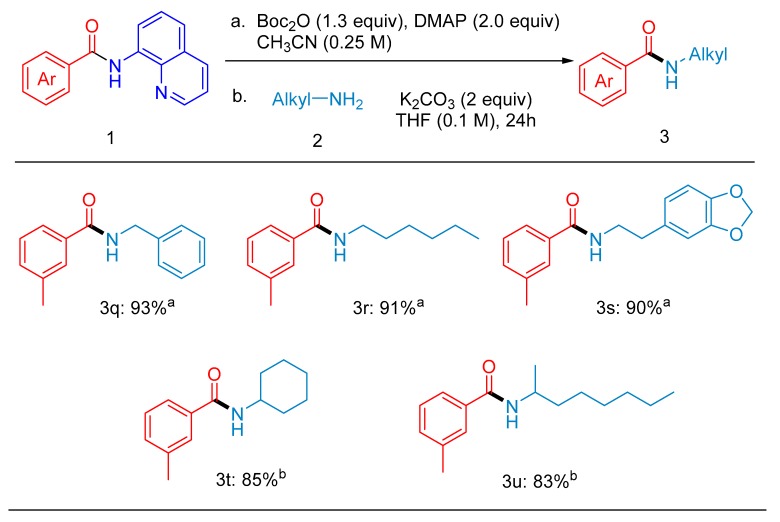
Scope of alkyl amines. ^a^ Reaction temperature 25 °C, ^b^ 80 °C.

Next, we tried to expand the scope to aliphatic amines (Figure 3). The reaction took place without Pd catalysts, which was consistent with Szostak’s report [66,76]. For the primary amines, the reaction proceeded well even at room temperature (**3p**, **3q**, **3r**). For the secondary amines, although a higher temperature was required, the yield was still excellent (**3s**, **3t**).

**Scheme 2:**
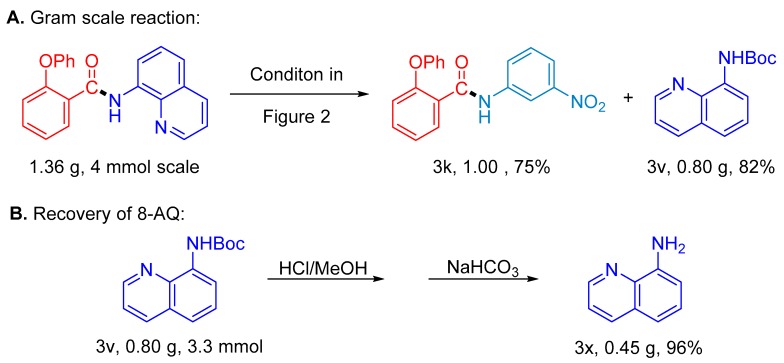
Gram-scale reaction and recovery of 8-AQ.

Finally, we scaled up the reaction to the gram scale (Scheme 2A) and found that the yield was consistent with the small-scale reactions. We also isolated the *tert*-butyl quinolin-8-ylcarbamate in 82% yield, from which the Boc deprotection can be removed quantitatively to recover the 8-AQ (Scheme 2B).

For the mechanism, we postulated that the amide bond was activated by Boc via a steric-induced geometry twist, which facilitated the oxidative addition of a Pd–NHC catalyst to generate an acylpalladium intermediate. The more nucleophilic amine then substituted the 8-AQ carbamate via ligand exchanges, which further underwent reductive elimination to form the product while regenerating the Pd(0) species.

## 3. Discussion

To summarize, we reported the Boc-activated transamidation of 8-AQ, which is a widely utilized auxiliary in C–H functionalization. Boc protection of the corresponding 8-AQ amide was critical to activate the amide C_(acyl)_–N bond by twisting its geometry and lowering the amidic resonance energy. Both aryl and alkyl amines proceeded transamidation in excellent yields. The facile and mild Boc activation of the amide C_(acyl)_–N bond as well as the user-friendly one-pot reaction procedure highlight its utilization in the future. This method provides alternative means to remove 8-AQ that is complementary to existing protocols. Future efforts will be directed toward expanding the types of new transformations as well as the different types of directing groups.

## 4. Experimental Section

### 4.1. Representative General Procedure for One-pot Transamidation of 8-Aminoquinoline Amides

8-aminoquinoline amide (0.2 mmol, 60.5 mg), di-*tert*-butyl-dicarbonate (52.4 mg, 1.2 equiv), *N,N-*dimethylpyridin-4-amine (26.9 mg, 1.1 equiv), and acetonitrile (2 mL, 0.1 M) were added to an oven-dried Schlenk pressure vessel equipped with a stir bar. The resulting reaction mixture was stirred at room temperature for 15 h. The solvent was removed under high vacuum after the indicated time. The same reaction vial was charged with aniline (28 mg, 0.3 mmol), potassium carbonate (55 mg, 2.0 equiv), and (IPr)Pd(cinnamyl)Cl (3.9 mg, 3 mol%) (not needed for alkyl amine), placed under a positive pressure of argon, and subjected to three evacuation/backfilling cycles under high vacuum. THF (2 mL, 0.1 M) was added with vigorous stirring at room temperature, and the reaction took place at the indicated temperate (aniline at 110 °C, primary amine at room temperature, and secondary amine at 80 °C) and was stirred for 24 h. The reaction mixture was cooled down to room temperature and diluted with ethyl acetate (10 mL) after the indicated time. The reaction mixture was filtered and concentrated. Purification by chromatography on silica gel (EtOAc/hexanes) afforded the product (yield: 88% (37.2 mg), white solid). Characterization data matched those described above.

### 4.2. Characterization Data for Products ***3a**–**3u*** (Figure 2 and Figure 3)

*3-Methyl-N-phenylbenzamide* (**3a**): White solid. ^1^H-NMR (400 MHz, Chloroform-*d*) δ 7.90 (s, 1H), 7.70–7.59 (m, 4H), 7.40–7.30 (m, 4H), 7.14 (t, *J* = 7.4 Hz, 1H), 2.41 (s, 3H). ^13^C-NMR (101 MHz, CDCl_3_) δ 166.0, 138.7, 138.0, 135.0, 132.5, 129.0, 128.6, 127.8, 124.5, 123.9, 120.2, 21.4.

*3-Methyl-N-(p-tolyl)benzamide* (**3b**): White solid. ^1^H-NMR (400 MHz, Chloroform-*d*) δ 7.77 (s, 1H), 7.68 (s, 1H), 7.63 (dt, *J* = 5.7, 2.2 Hz, 1H), 7.58–7.50 (m, 2H), 7.39–7.31 (dd, *J* = 5.0, 1.9 Hz, 2H), 7.17 (d, *J* = 8.1 Hz, 2H), 2.42 (s, 3H), 2.34 (s, 3H). ^13^C-NMR (101 MHz, CDCl_3_) δ 165.8, 138.6, 135.4, 135.1, 134.1, 132.4, 129.5, 128.6, 127.7, 123.9, 120.2, 21.4, 20.9.

*3-Methyl-N-(o-tolyl)benzamide* (**3c**): White solid. ^1^H-NMR (400 MHz, Chloroform-*d*) δ 7.93 (d, *J* = 8.0 Hz, 1H), 7.76–7.59 (m, 3H), 7.37 (d, *J* = 5.5 Hz, 2H), 7.29–7.19 (m, 2H), 7.11 (t, *J* = 7.5 Hz, 1H), 2.44 (s, 3H), 2.33 (s, 3H). ^13^C-NMR (101 MHz, CDCl_3_) δ 165.8, 138.8, 135.8, 135.0, 132.6, 130.5, 129.2, 128.6, 127.9, 126.9, 125.3, 123.9, 123.1, 21.4, 17.8.

*N-(4-Methoxyphenyl)-3-methylbenzamide* (**3d**): White solid. ^1^H-NMR (400 MHz, Chloroform-*d*) δ 7.69 (d, *J* = 8.8 Hz, 2H), 7.63 (d, *J* = 6.3 Hz, 1H), 7.58–7.50 (m, 2H), 7.40–7.31 (m, 2H), 6.95–6.86 (m, 2H), 3.82 (s, 3H), 2.43 (s, 3H). ^13^C-NMR (101 MHz, CDCl_3_) δ 166.0, 156.5, 138.5, 134.9, 132.3, 131.1, 128.4, 127.8, 124.0, 122.2, 114.1, 55.4, 21.3.

*N-(2-Methoxyphenyl)-3-methylbenzamide* (**3e**): White solid. ^1^H-NMR (400 MHz, Chloroform-*d*) δ 8.53 (d, *J* = 6.6 Hz, 2H), 7.75–7.69 (m, 1H), 7.66 (d, *J* = 6.8 Hz, 1H), 7.37 (d, *J* = 7.3 Hz, 2H), 7.12–6.96 (m, 2H), 6.92 (d, *J* = 7.9 Hz, 1H), 3.93 (s, 3H), 2.44 (s, 3H). ^13^C-NMR (101 MHz, CDCl_3_) δ 165.5, 148.1, 138.6, 135.3, 132.4, 128.6, 127.8, 127.8, 123.9, 123.8, 121.2, 119.8, 109.9, 55.8, 21.4.

*N-(2,5-Dimethoxyphenyl)-3-methylbenzamide* (**3f**): White solid. ^1^H-NMR (400 MHz, Chloroform-*d*) δ 8.56 (s, 1H), 8.29 (d, *J* = 3.0 Hz, 1H), 7.78–7.62 (m, 2H), 7.37 (d, *J* = 7.0 Hz, 2H), 6.83 (d, *J* = 8.9 Hz, 1H), 6.61 (dd, *J* = 8.9, 3.0 Hz, 1H), 3.88 (s, 3H), 3.82 (s, 3H), 2.44 (s, 3H). ^13^C-NMR (101 MHz, CDCl_3_) δ 165.4, 153.9, 142.3, 138.7, 135.2, 132.5, 128.6, 128.5, 127.8, 123.9, 110.7, 108.8, 105.9, 56.3, 55.8, 21.4.

*3-Methyl-N-(3-nitrophenyl)benzamide* (**3g**): Pale yellow solid. ^1^H-NMR (400 MHz, Chloroform-*d*) δ 8.49 (t, *J* = 2.1 Hz, 1H), 8.22 (s, 1H), 8.11 (d, *J* = 8.2 Hz, 1H), 7.98 (d, *J* = 8.2 Hz, 1H), 7.74–7.62 (m, 2H), 7.52 (t, *J* = 8.2 Hz, 1H), 7.37 (d, *J* = 5.3 Hz, 2H), 2.42 (s, 3H). ^13^C-NMR (101 MHz, CDCl_3_) δ 166.2, 148.6, 139.1, 138.9, 133.9, 133.2, 129.9, 128.8, 127.8, 125.9, 124.1, 119.0, 114.9, 21.3.

*3-Methyl-N-(naphthalen-1-yl)benzamide* (**3h**): White solid. ^1^H-NMR (400 MHz, Chloroform-*d*) δ 8.20 (s, 1H), 8.03 (d, *J* = 7.5 Hz, 1H), 7.90 (dt, *J* = 6.5, 2.9 Hz, 2H), 7.84–7.70 (m, 3H), 7.52 (td, *J* = 8.0, 7.3, 4.4 Hz, 3H), 7.41 (d, *J* = 6.0 Hz, 2H), 2.46 (s, 3H). ^13^C-NMR (101 MHz, CDCl_3_) δ 166.5, 138.6, 134.7, 134.1, 132.6, 132.4, 128.7, 128.6, 128.0, 127.6, 126.2, 126.0, 125.9, 125.7, 124.1, 121.4, 120.9, 21.3.

*2-Methyl-N-phenylbenzamide* (**3i**): Pale yellow oil. ^1^H-NMR (400 MHz, Chloroform-*d*) δ 7.65 (d, *J* = 7.9 Hz, 2H), 7.57 (s, 1H), 7.50 (d, *J* = 7.6 Hz, 1H), 7.39 (t, *J* = 8.0 Hz, 3H), 7.29 (t, *J* = 5.3 Hz, 2H), 7.18 (t, *J* = 7.5 Hz, 1H), 2.53 (s, 3H). ^13^C-NMR (101 MHz, CDCl_3_) δ 168.2, 138.0, 136.4, 131.2, 130.2, 129.1, 126.6, 125.9, 124.5, 119.9, 19.8.

*N-(4-Isopropoxyphenyl)-2-methylbenzamide* (**3j**): White solid. ^1^H-NMR (400 MHz, Chloroform-*d*) δ 7.84–7.61 (m, 3H), 7.54 (d, *J* = 8.4 Hz, 2H), 7.44–7.32 (m, 2H), 6.92 (d, *J* = 8.4 Hz, 2H), 4.63–4.47 (m, 1H), 2.45 (s, 3H), 1.36 (d, *J* = 6.0 Hz, 6H). ^13^C-NMR (101 MHz, CDCl_3_) δ 166.2, 154.7, 138.4, 134.9, 132.3, 131.1, 128.4, 127.9, 124.1, 122.3, 116.3, 70.2, 22.0, 21.3.

*N-(3-Nitrophenyl)-2-phenoxybenzamide* (**3k**): Yellow solid. ^1^H-NMR (400 MHz, Chloroform-*d*) δ 9.90 (s, 1H), 8.50 (d, *J* = 2.8 Hz, 1H), 8.35 (d, *J* = 7.9 Hz, 1H), 7.98 (dd, *J* = 14.1, 8.4 Hz, 2H), 7.47 (td, *J* = 10.7, 9.1, 5.7 Hz, 4H), 7.29 (d, *J* = 7.5 Hz, 2H), 7.16 (d, *J* = 8.0 Hz, 2H), 6.90 (d, *J* = 8.3 Hz, 1H). ^13^C-NMR (101 MHz, CDCl_3_) δ 163.1, 155.6, 154.9, 148.6, 139.2, 133.7, 132.6, 130.5, 129.7, 126.0, 125.4, 124.0, 123.0, 119.8, 118.9, 118.2, 115.1.

*N-(2-Fluorophenyl)-4-methylbenzamide* (**3l**): White solid. ^1^H-NMR (400 MHz, Chloroform-*d*) δ 8.40 (d, *J* = 15.6 Hz, 1H), 8.24–8.12 (m, 1H), 7.60–7.46 (m, 3H), 7.31 (t, *J* = 7.6 Hz, 1H), 7.23–7.12 (m, 3H), 2.35 (s, 3H). ^13^C-NMR (101 MHz, Chloroform-*d*) δ 161.2, 160.4 (d, *J* = 246.2 Hz), 135.1, 134.4, 133.6 (d, *J* = 9.4 Hz), 132.3 (d, *J* = 2.0 Hz), 129.5, 125.0 (d, *J* = 3.3 Hz), 121.4 (d, *J* = 11.1 Hz), 120.5, 116.1 (d, *J* = 25.1 Hz).

*N-Mesitylquinoline-2-carboxamide* (**3m**): White solid. ^1^H-NMR (400 MHz, Chloroform-*d*) δ 9.67 (s, 1H), 8.41 (q, *J* = 8.6 Hz, 2H), 8.20 (d, *J* = 8.5 Hz, 1H), 7.95 (d, *J* = 8.2 Hz, 1H), 7.83 (t, *J* = 7.8 Hz, 1H), 7.68 (t, *J* = 7.6 Hz, 1H), 6.99 (s, 2H), 2.34 (s, 3H), 2.32 (s, 6H). ^13^C-NMR (101 MHz, CDCl_3_) δ 162.7, 149.7, 146.5, 137.6, 136.8, 135.2, 131.2, 130.2, 129.8, 129.4, 128.9, 128.0, 127.8, 119.0, 21.0, 18.6.

*N-Phenylquinoline-3-carboxamide* (**3n**): White solid. ^1^H-NMR (400 MHz, Chloroform-*d*) δ 10.27 (s, 1H), 8.41 (q, *J* = 8.5 Hz, 2H), 8.22 (d, *J* = 8.5 Hz, 1H), 8.00–7.78 (m, 4H), 7.68 (t, *J* = 7.6 Hz, 1H), 7.45 (t, *J* = 7.8 Hz, 2H), 7.21 (t, *J* = 7.4 Hz, 1H). ^13^C-NMR (101 MHz, CDCl_3_) δ 162.1, 149.6, 146.3, 137.9, 137.8, 130.3, 129.7, 129.4, 129.1, 128.2, 127.8, 124.3, 119.7, 118.7.

*2-(4-Isobutylphenyl)-N-(4-methoxyphenyl)propenamide* (**3o**): White solid. ^1^H-NMR (400 MHz, Chloroform-*d*) δ 7.31 (d, *J* = 8.6 Hz, 2H), 7.26 (d, *J* = 7.2 Hz, 2H), 7.15 (d, *J* = 7.7 Hz, 2H), 6.97 (s, 1H), 6.80 (d, *J* = 8.6 Hz, 2H), 3.76 (s, 3H), 3.67 (q, *J* = 7.2 Hz, 1H), 2.47 (d, *J* = 7.1 Hz, 2H), 1.95–1.79 (m, 1H), 1.58 (d, *J* = 7.2 Hz, 3H), 0.91 (d, *J* = 6.6 Hz, 6H). ^13^C-NMR (101 MHz, CDCl_3_) δ 172.5, 156.3, 140.9, 138.2, 131.1, 129.7, 127.4, 121.6, 114.0, 55.4, 47.4, 45.0, 30.1, 22.3, 18.5.

*N-(4-Methoxyphenyl)-2-methyl-2-phenylpropanamide* (**3p**): White solid. ^1^H-NMR (400 MHz, DMSO-*d*_6_) δ 9.00 (s, 1H), 7.47 (d, *J* = 8.5 Hz, 2H), 7.42–7.30 (m, 4H), 7.24 (dt, *J* = 6.4, 3.1 Hz, 1H), 6.84 (d, *J* = 8.8 Hz, 2H), 3.70 (s, 3H), 1.55 (s, 6H). ^13^C-NMR (101 MHz, DMSO) δ 174.7, 155.7, 146.5, 132.7, 128.7, 126.7, 126.1, 122.3, 113.9, 55.5, 47.5, 27.3.

*N-Benzyl-3-methylbenzamide* (**3q**): White solid. ^1^H-NMR (400 MHz, Chloroform-*d*) δ 7.61 (s, 1H), 7.57 (s, 1H), 7.39–7.26 (m, 7H), 6.56 (s, 1H), 4.61 (d, *J* = 5.7 Hz, 2H), 2.37 (s, 3H). ^13^C-NMR (101 MHz, CDCl_3_) δ 167.6, 138.4, 138.3, 134.3, 132.2, 128.7, 128.4, 127.9, 127.7, 127.5, 123.9, 44.1, 21.3.

*N-Hexyl-3-methylbenzamide* (**3r**): White solid. ^1^H-NMR (400 MHz, Chloroform-*d*) δ 7.51 (s, 1H), 7.49–7.42 (m, 1H), 7.20 (d, *J* = 4.7 Hz, 2H), 6.30 (s, 1H), 3.44–3.27 (m, 2H), 2.29 (s, 3H), 1.58–1.44 (m, 2H), 1.32–1.16 (m, 6H), 0.87–0.75 (m, 3H). ^13^C-NMR (101 MHz, CDCl_3_) δ 167.8, 138.2, 134.8, 131.9, 128.3, 127.7, 123.8, 40.1, 31.5, 29.6, 26.6, 22.5, 21.3, 14.0.

*N-(Benzo[d][1,3]dioxol-5-yl)-3-methylbenzamide* (**3s**): White solid. ^1^H-NMR (400 MHz, Chloroform-*d*) δ 7.54 (s, 1H), 7.49–7.42 (m, 1H), 7.28–7.24 (m, 2H), 6.81–6.60 (m, 3H), 6.26 (s, 1H), 3.64 (q, *J* = 6.6 Hz, 2H), 2.83 (t, *J* = 6.9 Hz, 2H), 2.37 (s, 3H). ^13^C-NMR (101 MHz, CDCl_3_) δ 167.7, 147.9, 146.2, 138.4, 134.6, 132.6, 132.1, 128.4, 127.6, 123.7, 121.7, 109.1, 108.3, 100.9, 41.3, 35.4, 21.3.

*N-Cyclohexyl-3-methylbenzamide* (**3t**): White solid. ^1^H-NMR (400 MHz, Chloroform-*d*) δ 7.57 (s, 1H), 7.55–7.48 (m, 1H), 7.35–7.27 (m, 2H), 5.93 (s, 1H), 4.05–3.90 (m, 1H), 2.40 (s, 3H), 2.10–1.96 (m, 2H), 1.84–1.56 (m, 4H), 1.52–1.35 (m, 2H), 1.29–1.18 (m, 2H). ^13^C-NMR (101 MHz, CDCl_3_) δ 166.8, 138.2, 135.1, 131.8, 128.3, 127.6, 123.8, 48.6, 33.1, 25.5, 24.9, 21.3.

*3-Methyl-N-(octan-2-yl)benzamide* (**3u**): Colorless oil. ^1^H-NMR (400 MHz, Chloroform-*d*) δ 7.59 (s, 1H), 7.54 (t, *J* = 4.6 Hz, 1H), 7.26 (d, *J* = 4.6 Hz, 2H), 6.53 (s, 1H), 3.36 (td, *J* = 6.2, 2.0 Hz, 2H), 2.35 (s, 3H), 1.65–1.48 (q, *J* = 6.1 Hz, 1H), 1.42–1.23 (m, 8H), 0.98–0.80 (m, 6H). ^13^C-NMR (101 MHz, CDCl_3_) δ 167.9, 138.2, 134.9, 131.9, 128.3, 127.7, 123.8, 43.0, 39.4, 31.0, 28.9, 24.3, 23.0, 21.3, 14.0, 10.8.

*Tert-Butyl quinolin-8-ylcarbamate* (**3v**): Brown solid. ^1^H-NMR (400 MHz, Chloroform-*d*) δ 9.05 (s, 1H), 8.82 (dd, *J* = 4.1, 2.1 Hz, 1H), 8.44 (d, *J* = 7.6 Hz, 1H), 8.16 (d, *J* = 8.3 Hz, 1H), 7.54 (t, *J* = 8.0 Hz, 1H), 7.45 (d, *J* = 7.8 Hz, 2H), 1.60 (s, 9H).^13^C-NMR (101 MHz, CDCl_3_) δ 152.94, 148.00, 138.24, 136.30, 135.18, 128.07, 127.38, 121.56, 120.18, 114.42, 80.45, 28.43.

*8-Aminequinoline* (**3x**): Pale yellow solid. ^1^H-NMR (400 MHz, DMSO-*d*_6_) δ 8.73 (dt, *J* = 3.7, 1.8 Hz, 1H), 8.18 (dt, *J* = 8.3, 1.8 Hz, 1H), 7.46 (ddd, *J* = 8.3, 4.2, 1.9 Hz, 1H), 7.30 (td, *J* = 7.9, 1.8 Hz, 1H), 7.06 (dd, *J* = 8.1, 2.2 Hz, 1H), 6.87 (d, *J* = 7.6 Hz, 1H), 5.94 (s, 2H). ^13^C-NMR (101 MHz, DMSO) δ 147.43, 145.68, 137.84, 136.31, 129.02, 128.07, 121.91, 114.12, 109.09.

## 5. Conclusions

In summary we reported a mild, one-pot Pd catalyzed transamidation of 8-aminoquinoline. The mind and facile Boc protection activate the amide C_(acyl)_–N bond by twisting its geometry to lower the amidic resonance energy for facile Pd oxidative addition. This strategy gives a new choice in removal of directing groups after the C-H functionalization.

## Figures and Tables

**Table 1:** Optimization of removal of 8-AQ.

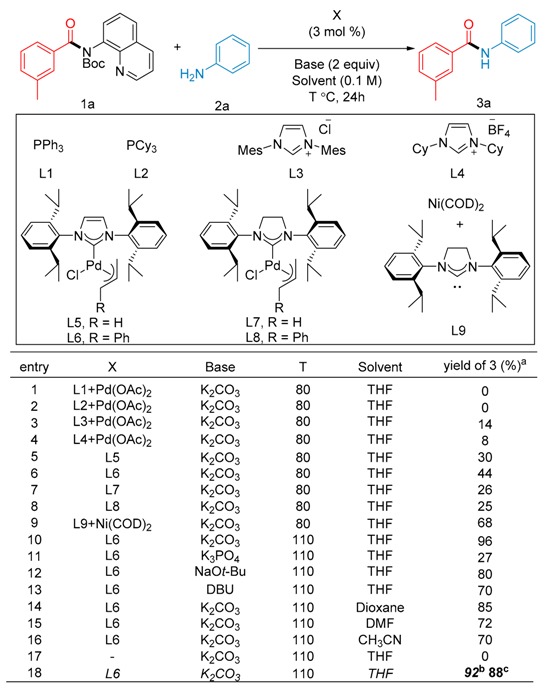

^a^ Conditions: amide (0.2 mmol), amine (1.5 equiv), yield determined by mesitylene as an internal standard. ^b^ 8-AQ amide (0.2 mmol), 4-dimethylaminopyridine (DMAP) (1.1 equiv), di-*tert*-butyl decarbonate (Boc_2_O) (1.2 equiv), and CH_3_CN (0.1 M) were reacted for 12 h. ^c^ Isolated yield.

## Supplementary Materials

Supplementary Materials are available online.

## References

[1] Shilov A.E., Shul’pin G.B. (1997). Activation of C-H bonds by metal complexes. Chem. Rev..

[2] He J., Wasa M., Chan K.S.L., Shao Q., Yu J.-Q. (2017). Palladium Catalyzed Transformations of Alkyl C−H Bonds. Chem. Rev..

[3] Park Y., Kim Y., Chang S. (2017). Transition Metal-Catalyzed C−H Amination: Scope, Mechanism, and Applications. Chem. Rev..

[4] Dong Z., Ren Z., Thompson S.J., Xu Y., Dong G. (2017). Transition-Metal-Catalyzed C−H Alkylation Using Alkenes. Chem. Rev..

[5] Chen M.S., White M.C. (2007). A predictably selective aliphatic C-H oxidation reaction for complex molecule synthesis. Science.

[6] Qin Y., Zhu L., Luo S. (2017). Organocatalysis in Inert C−H Bond Functionalization. Chem. Rev..

[7] Abrams D.J., Provenchera P.A., Sorensen E.J. (2018). Recent applications of C–H functionalization in complex natural product synthesis. Chem. Soc. Rev..

[8] Shi H., Herron A.N., Shao Y., Shao Q., Yu J.-Q. (2018). Enantioselective remote meta-C-H arylation and alkylation via a chiral transient mediator. Nature.

[9] Murai S., Kakiuchi F., Sekine S., Tanaka Y., Kamatani A., Sonoda M., Chatani N. (1993). Efficient catalytic addition of aromatic carbon-hydrogen bonds to olefins. Nature.

[10] Kimura N., Kochi T., Kakiuchi F. (2017). Iron-Catalyzed Regioselective Anti-Markovnikov Addition of C–H Bonds in Aromatic Ketones to Alkenes. J. Am. Chem. Soc..

[11] Xiao B., Gong T.-J., Xu J., Liu Z.-J., Liu L. (2011). Palladium-Catalyzed Intermolecular Directed C−H Amidation of Aromatic Ketones. J. Am. Chem. Soc..

[12] Xiao B., Fu Y., Xu J., Gong T.-J., Dai J.-J., Yi J., Liu L. (2010). Pd(II)-Catalyzed C−H Activation/Aryl−Aryl Coupling of Phenol Esters. J. Am. Chem. Soc..

[13] Trost B.M., Imi K., Davies I.W. (1995). Elaboration of Conjugated Alkenes Initiated by Insertion into a Vinylic C-H Bond. J. Am. Chem. Soc..

[14] Li G., Wan L., Zhang G., Leow D., Spangler J., Yu J.-Q. (2015). Pd(II)-Catalyzed C–H Functionalizations Directed by Distal Weakly Coordinating Functional Groups. J. Am. Chem. Soc..

[15] Huang L., Hackenberger D., Goossen L.J. (2015). Iridium-Catalyzed ortho-Arylation of Benzoic Acids with Arenediazonium Salts. Angew. Chem. Int. Ed..

[16] Biafora A., Krause T., Hackenberger D., Belitz F., Goossen L.J. (2016). *ortho*-C−H Arylation of Benzoic Acids with Aryl Bromides and Chlorides Catalyzed by Ruthenium. Angew. Chem. Int. Ed..

[17] Dastbaravardeh N., Toba T., Farmer M.E., Yu J.-Q. (2015). Monoselective o-C–H Functionalizations of Mandelic Acid and α-Phenylglycine. J. Am. Chem. Soc..

[18] Gao P., Guo W., Xue J.J., Zhao Y., Yuan Y., Xia Y.Z., Shi Z.Z. (2015). Iridium(III)-Catalyzed Direct Arylation of C-H Bonds with Diaryliodonium Salts. J. Am. Chem. Soc..

[19] Yang W., Ye S., Fanning D., Coon T., Schmidt Y., Krenitsky P., Stamos D., Yu J.-Q. (2015). Orchestrated Triple C-H Activation Reactions Using Two Directing Groups: Rapid Assembly of Complex Pyrazoles. Angew. Chem. Int. Ed..

[20] Boerth J.A., Ellman J.A. (2017). A Convergent Synthesis of Functionalized Alkenyl Halides through Cobalt(III)-Catalyzed Three-Component C–H Bond Addition. Angew. Chem. Int. Ed..

[21] Ikemoto H., Kanai M., Tanaka R., Yoshino T., Matsunaga S., Sakata K., Sakata K., Yoshino T., Matsunaga S. (2017). Stereoselective Synthesis of Tetrasubstituted Alkenes via a Cp*CoIII-Catalyzed C–H Alkenylation/Directing Group Migration Sequence. Angew. Chem. Int. Ed..

[22] Tran L.D., Daugulis O. (2012). Nonnatural Amino Acid Synthesis by Using Carbon–Hydrogen Bond Functionalization Methodology. Angew. Chem. Int. Ed..

[23] Zaitsev V.G., Shabashov D., Daugulis O. (2005). Highly Regioselective Arylation of sp^3^ C−H Bonds Catalyzed by Palladium Acetate. J. Am. Chem. Soc..

[24] Feng Y., Chen G. (2010). Total Synthesis of Celogentin C by Stereoselective C–H Activation. Angew. Chem. Int. Ed..

[25] Shabashov D., Daugulis O. (2010). Auxiliary-Assisted Palladium-Catalyzed Arylation and Alkylation of sp^2^ and sp^3^ Carbon−Hydrogen Bonds. J. Am. Chem. Soc..

[26] Ano Y., Tobisu M., Chatani N. (2011). Palladium-Catalyzed Direct Ethynylation of C(sp^3^)–H Bonds in Aliphatic Carboxylic Acid Derivatives. J. Am. Chem. Soc..

[27] Gutekunst W.R., Gianatassio R., Baran P.S. (2012). Sequential Csp^3^–H Arylation and Olefination: Total Synthesis of the Proposed Structure of Pipercyclobutanamide A. Angew. Chem. Int. Ed..

[28] Shibata K., Chatani N. (2016). Rhodium-catalyzed regioselective addition of the *ortho* C–H bond in aromatic amides to the C–C double bond in α,β-unsaturated γ-lactones and dihydrofurans. Chem. Sci..

[29] Shibata K., Natsui S., Chatani N. (2017). Rhodium-Catalyzed Alkenylation of C–H Bonds in Aromatic Amides with Alkynes. Org. Lett..

[30] Yang Y., Hou W., Qin L., Du J., Feng H., Zhou B., Li Y. (2014). Rhodium-Catalyzed Directed Sulfenylation of Arene C–H Bonds. Chem.–Eur. J..

[31] Shan C., Luo X., Qi X., Liu S., Li Y., Lan Y. (2016). Mechanism of Ruthenium-Catalyzed Direct Arylation of C–H Bonds in Aromatic Amides: A Computational Study. Organometallics.

[32] Aihara Y., Tobisu M., Fukumoto Y., Chatani N. (2014). Ni(II)-Catalyzed Oxidative Coupling between C(sp^2^)–H in Benzamides and C(sp^3^)–H in Toluene Derivatives. J. Am. Chem. Soc..

[33] Aihara Y., Chatani N. (2014). Nickel-Catalyzed Direct Arylation of C(sp^3^)–H Bonds in Aliphatic Amides via Bidentate-Chelation Assistance. J. Am. Chem. Soc..

[34] Aihara Y., Chatani N. (2013). Nickel-Catalyzed Direct Alkylation of C–H Bonds in Benzamides and Acrylamides with Functionalized Alkyl Halides via Bidentate-Chelation Assistance. J. Am. Chem. Soc..

[35] Wu X., Zhao Y., Ge H. (2015). Direct Aerobic Carbonylation of C(sp^2^)–H and C(sp^3^)–H Bonds through Ni/Cu Synergistic Catalysis with DMF as the Carbonyl Source. J. Am. Chem. Soc..

[36] Wu X., Zhao Y., Ge H. (2014). Nickel-Catalyzed Site-Selective Alkylation of Unactivated C(sp^3^)–H Bonds. J. Am. Chem. Soc..

[37] Yi J., Yang L., Xia C., Li F. (2015). Nickel-Catalyzed Alkynylation of a C(sp^2^)–H Bond Directed by an 8-Aminoquinoline Moiety. J. Org. Chem..

[38] Kubo T., Chatani N. (2016). Dicumyl Peroxide as a Methylating Reagent in the Ni-Catalyzed Methylation of Ortho C–H Bonds in Aromatic Amides. Org. Lett..

[39] Zhang J., Li D., Chen H., Wang B., Liu Z., Zhang Y. (2016). Copper(II)/Silver(I)-Catalyzed Sequential Alkynylation and Annulation of Aliphatic Amides with Alkynyl Carboxylic Acids: Efficient Synthesis of Pyrrolidones. Adv. Synth. Catal..

[40] Takamatsu K., Hirano K., Masahiro M. (2017). Copper-Mediated Decarboxylative Coupling of Benzamides with *ortho-*Nitrobenzoic Acids by Directed C–H Cleavage. Angew. Chem. Int. Ed..

[41] Liu J., Yu L., Zhuang S., Gui Q., Chen X., Wang W., Tan Z. (2015). Copper-mediated *ortho* C–H sulfonylation of benzoic acid derivatives with sodium sulfinates. Chem. Commun..

[42] Williamson P., Galvan A., Gaunt M.J. (2017). Cobalt-catalysed C–H carbonylative cyclisation of aliphatic amides. Chem. Sci..

[43] Zhang J., Chen H., Lin C., Liu Z., Wang C., Zhang Y. (2015). Cobalt-Catalyzed Cyclization of Aliphatic Amides and Terminal Alkynes with Silver-Cocatalyst. J. Am. Chem. Soc..

[44] Zhang Z.-Z., Han Y.-Q., Zhan B.-B., Wang S., Shi B.-F. (2017). Synthesis of Bicyclo[*n*.1.0]alkanes by a Cobalt-Catalyzed Multiple C(sp^3^)–H Activation Strategy. Angew. Chem. Int. Ed..

[45] Shang R., Ilies L., Nakamura E. (2015). Iron-Catalyzed Directed C(sp^2^)–H and C(sp^3^)–H Functionalization with Trimethylaluminum. J. Am. Chem. Soc..

[46] Shang R., Ilies L., Asako S., Nakamura E. (2014). Iron-Catalyzed C(sp^2^)–H Bond Functionalization with Organoboron Compounds. J. Am. Chem. Soc..

[47] Ilies L., Matsubara T., Ichikawa S., Asako S., Nakamura E. (2014). Iron-Catalyzed Directed Alkylation of Aromatic and Olefinic Carboxamides with Primary and Secondary Alkyl Tosylates, Mesylates, and Halides. J. Am. Chem. Soc..

[48] Shang R., Ilies R., Matsumoto A., Nakamura E. (2013). β-Arylation of Carboxamides via Iron-Catalyzed C(sp^3^)–H Bond Activation. J. Am. Chem. Soc..

[49] Trost B.M., Fleming I. (1991). Comprehensive Organic Synthesis.

[50] Zhang S.-Y., Li Q., He G., Nack W.A., Chen G. (2013). Stereoselective Synthesis of -Alkylated -Amino Acids via Palladium-Catalyzed Alkylation of Methylene C(sp^3^)−H Bonds. J. Am. Chem. Soc..

[51] Deguchi T., Xin H.-L., Morimoto H., Ohshima T. (2017). Direct Catalytic Alcoholysis of Unactivated 8-Aminoquinoline Amides. ACS Catal..

[52] He G., Zhang S.-Y., Nack W.A., Chen G. (2013). Use of a Readily Removable Auxiliary Group for the Synthesis of Pyrrolidones by the Palladium-Catalyzed Intramolecular Amination of Unactivated C(sp^3^)−H Bonds. Angew. Chem. Int. Ed..

[53] Spletstoser J.T., White J.M., Tunoori A.R., Georg G.I. (2007). Mild and Selective Hydrozirconation of Amides to Aldehydes Using Cp_2_Zr(H)Cl:  Scope and Mechanistic Insight. J. Am. Chem. Soc..

[54] Boit T.B., Weires N.A., Kim J., Garg N.K. (2018). Nickel-Catalyzed Suzuki–Miyaura Coupling of Aliphatic Amides. ACS Catal..

[55] Dander J.E., Baker E.L., Garg N.K. (2017). Nickel-Catalyzed Transamidation of Aliphatic Amide Derivatives. Chem. Sci..

[56] Weires N.A., Caspi D.D., Garg N.K. (2017). Kinetic Modeling of the Nickel-Catalyzed Esterification of Amides. ACS Catal..

[57] Medina J.M., Moreno J., Racine S., Du S., Garg N.K. (2017). Mizoroki–Heck Cyclizations of Amide Derivatives for the Introduction of Quaternary Centers. Angew. Chem. Int. Ed..

[58] Dander J.E., Garg N.K. (2017). Breaking Amides Using Nickel Catalysis. ACS Catal..

[59] Hie L., Baker E.L., Anthony S.M., Desrosiers J.-N., Senanayake C., Garg N.K. (2016). Nickel-Catalyzed Esterification of Aliphatic Amides. Angew. Chem. Int. Ed..

[60] Dander J.E., Weires N.A., Garg N.K. (2016). Benchtop Delivery of Ni(cod)_2_ using Paraffin Capsules. Org. Lett..

[61] Baker E.L., Yamano M.M., Zhou Y., Anthony S.M., Garg N.K. (2016). A Two-Step Approach to Achieve Secondary Amide Transamidation Enabled by Nickel Catalysis. Nat. Commun..

[62] DerivativesSimmons B.J., Weires N.A., Dander J.E., Garg N.K. (2016). Nickel-Catalyzed Alkylation of Amide. ACS Catal..

[63] Weires N.A., Baker E.L., Garg N.K. (2016). Nickel-Catalysed Suzuki–Miyaura Coupling of Amides. Nat. Chem..

[64] Hie L., Nathel N.F.F., Shah T.K., Baker E.L., Hong X., Yang Y.-F., Liu P., Houk K.N., Garg N.K. (2015). Conversion of Amides to Esters by the Nickel-Catalysed Activation of Amide C–N Bonds. Nature.

[65] Ra Liu C., Shi S., Liu Y., Liu R., Lalancette R., Szostak R., Szostak M. (2018). The Most Twisted Acyclic Amides: Structures and Reactivity. Org. Lett..

[66] Shi S., Nolan S.P., Szostak M. (2018). Well-Defined Palladium(II)-NHC (NHC = N-Heterocyclic Carbene) Precatalysts for Cross-Coupling Reactions of Amides and Esters by Selective Acyl CO–X (X = N, O) Cleavage. Acc. Chem. Res..

[67] Li G., Szostak M. (2018). Highly Selective Transition-Metal-Free Transamidation of Amides and Amidation of Esters at Room Temperature. Nature Commun..

[68] Liu C., Li G., Shi S., Meng G., Lalancette R., Szostak R., Szostak M. (2018). Acyl and Decarbonylative Suzuki Coupling of N-Acetyl Amides: Electronic Tuning of Twisted, Acyclic Amides in Catalytic Carbon−Nitrogen Bond Cleavage. ACS Catal..

[69] Li G., Lei P., Szostak M. (2018). Transition-Metal-Free Esterification of Amides via Selective N−C Cleavage under Mild Conditions. Org. Lett..

[70] Szostak R., Szostak M. (2018). N-Acyl-Glutarimides: Resonance and Proton Affinities of Rotationally-Inverted Twisted Amides Relevant to N−C(O) Cross-Coupling. Org. Lett..

[71] Meng G., Shi S., Lalancette R., Szostak R., Szostak M. (2018). Reversible Twisting of Primary Amides via Ground State N−C(O) Destabilization: Highly Twisted Rotationally Inverted Acyclic Amides. J. Am. Chem. Soc..

[72] Meng G., Szostak M. (2017). Site-Selective C−H/C−N Activation by Cooperative Catalysis: Primary Amides as Arylating Reagents in Directed C−H Arylation. ACS Catal..

[73] Shi S., Szostak M. (2017). Pd–PEPPSI: A General Pd–NHC Precatalyst for Buchwald–Hartwig Cross-Coupling of Esters and Amides (Transamidation) under the Same Reaction Conditions. Chem. Commun..

[74] Lei P., Meng G., Shi S., Ling Y., An J., Szostak R., Szostak M. (2017). Suzuki–Miyaura Cross-Coupling of Amides and Esters at Room Temperature: Correlation with Barriers to Rotation around C–N and C–O Bonds. Chem. Sci..

[75] Meng G., Szostak R., Szostak M. (2017). Suzuki−Miyaura Cross-Coupling of N-Acylpyrroles and Pyrazoles: Planar, Electronically Activated Amides in Catalytic N–C Cleavage. Org. Lett..

[76] Meng G., Lei P., Szostak M. (2017). A General Method for Two-Step Transamidation of Secondary Amides Using Commercially Available, Air- and Moisture-Stable Palladium/NHC (N-Heterocyclic Carbene) Complexes. Org. Lett..

[77] Liu Y., Shi S., Achtenhagen M., Liu R., Szostak M. (2017). Metal-Free Transamidation of Secondary Amides via Selective N–C Cleavage under Mild Conditions. Org. Lett..

[78] Lei P., Meng G., Szostak M. (2017). General Method for the Suzuki-Miyaura Cross-Coupling of Amides Using Commercially Available, Air- and Moisture-Stable Palladium/NHC (NHC = N-Heterocyclic Carbene) Complexes. ACS Catal..

[79] Shi S., Szostak M. (2016). Nickel-Catalyzed Diaryl Ketone Synthesis by N–C Cleavage: Direct Negishi Cross-Coupling of Primary Amides by Site- Selective *N*,*N*-Di-Boc Activation. Org. Lett..

[80] Meng G., Shi S., Szostak M. (2016). Palladium-Catalyzed Suzuki−Miyaura Cross-Coupling of Amides via Site-Selective N−C Bond Cleavage by Cooperative Catalysis. ACS Catal..

[81] Pace V., Holzer W., Meng G., Shi S., Lalancette R., Szostak R., Szostak M. (2016). Structures of Highly Twisted Amides Relevant to Amide N−C Cross-Coupling: Evidence for Ground-State Amide Destabilization. Chem. Eur. J..

[82] Shi S., Meng G., Szostak M. (2016). Synthesis of Biaryls via Nickel Catalyzed Suzuki–Miyaura Coupling of Amides by Carbon–Nitrogen Cleavage. Angew. Chem. Int. Ed..

[83] Meng G., Szostak M. (2015). Sterically-Controlled Pd-Catalyzed Chemoselective Ketone Synthesis via N–C Cleavage in Twisted Amides. Org. Lett..

[84] Di Gioia M.L., Gagliardi A., Leggio A., Leotta V., Romio E., Liguori A. (2015). N-Urethane protection of amines and amino acids in an ionic liquid. RSC Adv..

[85] Nardi M., Cano N.H., Costanzo P., Oliverio M., Sindona G., Procopio A. (2015). Aqueous MW eco-friendly protocol for amino group protection. RSC Adv..

[86] Durga T.V., Rambabu A., Reddy M.S., Babu B.H. (2017). Ionic Liquid as Efficient Reaction Medium for N-tert-Boc Protection of Amines. Asian J. Chem..

[87] Sunitha S., Kanjilal S., Reddy P.S., Prasad R.B.N. (2008). An efficient and chemoselective Brønsted acidic ionic liquid-catalyzed N-Boc protection of amines. Tetrahedron Lett..

[88] Schwarzer M.C., Konno R., Hojo T., Ohtsuki A., Nakamura K., Yasutome A., Takahashi H., Shimasaki T., Tobisu M., Chatani N. (2017). Combined Theoretical and Experimental Studies of Nickel-Catalyzed Cross-Coupling of Methoxyarenes with Arylboronic Esters via C–O Bond Cleavage. J. Am. Chem. Soc..

[89] Schaub T., Fischer P., Steffen A., Braun T., Radius U., Mix A. (2008). C−F Activation of Fluorinated Arenes using NHC-Stabilized Nickel(0) Complexes: Selectivity and Mechanistic Investigations. J. Am. Chem. Soc..

