# Supplementary material for: An Efficient, One-Pot Transamidation of 8-Aminoquinoline Amides Activated by Tertiary-Butyloxycarbonyl
Source: Molecules. 2019 Mar 29;24(7):1234. doi: 10.3390/molecules24071234 (PMC6480081; doi:10.3390/molecules24071234)
Supplement: Supplementary file 1 [file molecules-24-01234-s001.pdf]

Electronic Supplementary Information (ESI)

An Efficient, One-Pot Transamidation of 8-Aminoquinoline Amides Activated by Boc

Wengang Wu [1], Jun Yi [2,*], Huipeng Xu [2], Shuangjun Li [2] and Rongxin Yuan [2,*]

1 Soochow University, 199 Ren'ai Road, Suzhou, Jiangsu 215123, China
E-mail: 20155209006@suda.edu.cn (W. W.).
2 Jiangsu Laboratory of Advanced Functional Material, School of Chemistry and Materials Engineering, Changshu Institute of Technology, Changshu 215500, China

General Methods

Reagent-grade solvents were used for extraction, recrystallization, and flash chromatography. All other commercial reagents were used as received without additional purification. The progress of reactions was checked by analytical thin-layer chromatography (TLC, silica gel 60 F-254 plates) (Qingdao Haiyang Chemical Co., Ltd., Qingdao, Shandong, China). The plates were visualized first with UV illumination, followed by iodine or phosphomolybdic acid hydrate. Column chromatography was performed using silica gel (200–300 mesh) (Qingdao Haiyang Chemical Co., Ltd., Qingdao, Shandong, China). NMR spectra were obtained using a BRUKER AVANCE III instrument (Bruker Co., Ltd., Switzerland). ^{1}H-NMR spectra were recorded at 400 MHz and are reported in parts per million (ppm) on the δ scale relative to tetramethylsilane (TMS) as an internal standard. ^{13}C-NMR spectra were recorded at 100 MHz and are reported in parts per million (ppm) on the δ scale relative to $CDCl_3$ (δ 77.16). Multiplicities are indicated as the following: s, singlet; d, doublet; t, triplet; q, quartet; m, multiplet; dd, doubled doublet; td, tripled doublet; br, broad. Coupling constants (J values), where noted, are quoted in hertz. Mass spectra were obtained using an Agilent 1260-6120 (ESI) instrument (Agilent Technologies Co., Ltd., Santa Clara, CA, USA).

Supplementary Table 1. Screening of catalyst[a].

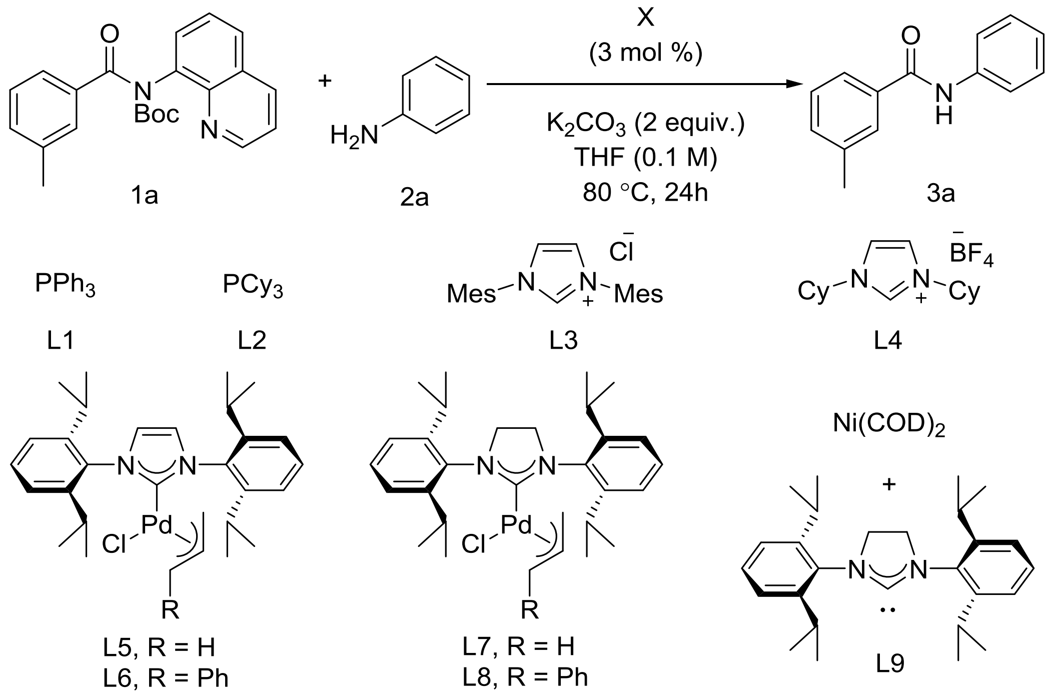

entry	X	yield of 3 (%)[a]
1	L1+Pd(OAc)$_2$	0
2	L2+Pd(OAc)$_2$	0
3	L3+Pd(OAc)$_2$	14
4	L4+Pd(OAc)$_2$	8
5	L5	30
6	L6	44
7	L7	26
8	L8	25
9	L9+Ni(COD)$_2$	68

[a]Reaction conditions (unless otherwise specified): 1a (0.2 mmol, 1.0 equiv), 2a (0.3 mmol, 1.5 equiv), and THF (2 mL). [b]Determined by mesitylene as an internal standard.

Supplementary Table 2. Screening of reaction temperature[a].

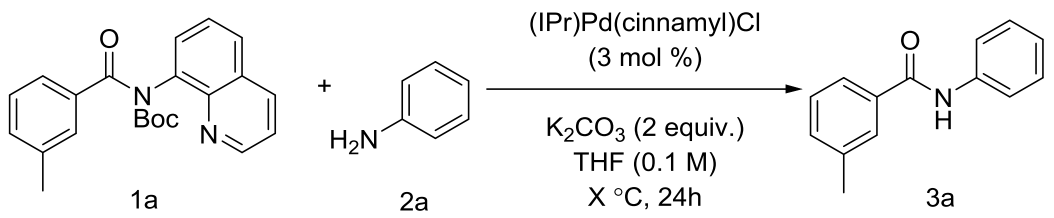

entry	X	yield of 3 (%)[a]
1	40	8
2	80	44
3	110	96
4	130	97

[a]Reaction conditions (unless otherwise specified): 1a (0.2 mmol, 1.0 equiv), 2a (0.3 mmol, 1.5 equiv), and THF (2 mL). [b]Determined by mesitylene as an internal standard.

Supplementary Table 3. Screening of solvent[a].

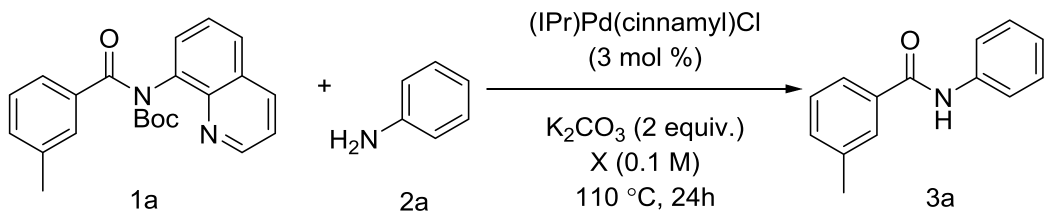

entry	X	yield of 3 (%)[a]
1	CH_2Cl_2	27
2	THF	96
3	DMF	72
4	DMAc	80
5	1, 4 - Dioxane	85
6	CH_3CN	70

[a]Reaction conditions (unless otherwise specified): 1a (0.2 mmol, 1.0 equiv), 2a (0.3 mmol, 1.5 equiv), and solvent (2 mL). [b]Determined by mesitylene as an internal standard.

Supplementary Table 4. Screening of base[a].

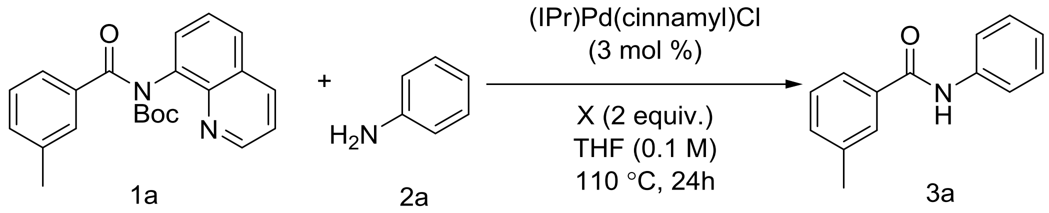

entry	X	yield of 3 (%)[a]
1	K_3PO_4	27
2	K_2CO_3	96
3	NaOH	72
4	*t*BuONa	80
5	Cs_2CO_3	85
6	DBU	70

[a]Reaction conditions (unless otherwise specified): 1a (0.2 mmol, 1.0 equiv), 2a (0.3 mmol, 1.5 equiv), and solvent (2 mL). [b]Determined by mesitylene as an internal standard.

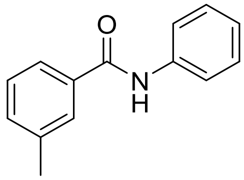

3-methyl-*N*-phenylbenzamide (3a):

^{1}H NMR (400 MHz, Chloroform-*d*) δ 7.90 (s, 1H), 7.70 – 7.59 (m, 4H), 7.40 – 7.30 (m, 4H), 7.14 (t, *J* = 7.4 Hz, 1H), 2.41 (s, 3H).

^{13}C NMR (101 MHz, $CDCl_3$) δ 166.0, 138.7, 138.0, 135.0, 132.5, 129.0, 128.6, 127.8, 124.5, 123.9, 120.2, 21.4.

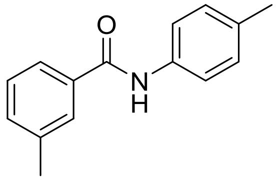

3-methyl-*N*-(p-tolyl)benzamide (3b):

^{1}H NMR (400 MHz, Chloroform-*d*) δ 7.77 (s, 1H), 7.68 (s, 1H), 7.63 (dt, *J* = 5.7, 2.2 Hz, 1H), 7.58 – 7.50 (m, 2H), 7.39 – 7.31 (dd, *J* = 5.0, 1.9 Hz, 2H), 7.17 (d, *J* = 8.1 Hz, 2H), 2.42 (s, 3H), 2.34 (s, 3H).

^{13}C NMR (101 MHz, $CDCl_3$) δ 165.8, 138.6, 135.4, 135.1, 134.1, 132.4, 129.5, 128.6, 127.7, 123.9, 120.2, 21.4, 20.9.

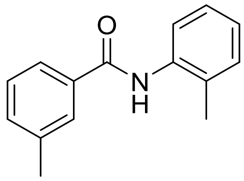

3-methyl-*N*-(o-tolyl)benzamide (3c):

^{1}H NMR (400 MHz, Chloroform-*d*) δ 7.93 (d, *J* = 8.0 Hz, 1H), 7.76 – 7.59 (m, 3H), 7.37 (d, *J* = 5.5 Hz, 2H), 7.29 – 7.19 (m, 2H), 7.11 (t, *J* = 7.5 Hz, 1H), 2.44 (s, 3H), 2.33 (s, 3H).

^{13}C NMR (101 MHz, $CDCl_3$) δ 165.8, 138.8, 135.8, 135.0, 132.6, 130.5, 129.2, 128.6, 127.9, 126.9, 125.3, 123.9, 123.1, 21.4, 17.8.

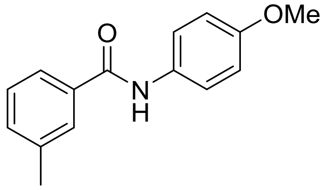

N-(4-methoxyphenyl)-3-methylbenzamide (3d):

^{1}H NMR (400 MHz, Chloroform-*d*) δ 7.69 (d, *J* = 8.8 Hz, 2H), 7.63 (d, *J* = 6.3 Hz, 1H), 7.58 – 7.50 (m, 2H), 7.40 – 7.31 (m, 2H), 6.95 – 6.86 (m, 2H), 3.82 (s, 3H), 2.43 (s, 3H).

^{13}C NMR (101 MHz, $CDCl_3$) δ 166.0, 156.5, 138.5, 134.9, 132.3, 131.1, 128.4, 127.8, 124.0, 122.2, 114.1, 55.4, 21.3.

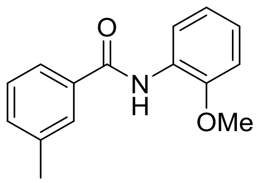

N-(2-methoxyphenyl)-3-methylbenzamide (3e):

^{1}H NMR (400 MHz, Chloroform-*d*) δ 8.53 (d, *J* = 6.6 Hz, 2H), 7.75 – 7.69 (m, 1H), 7.66 (d, *J* = 6.8 Hz, 1H), 7.37 (d, *J* = 7.3 Hz, 2H), 7.12 – 6.96 (m, 2H), 6.92 (d, *J* = 7.9 Hz, 1H), 3.93 (s, 3H), 2.44 (s, 3H).

^{13}C NMR (101 MHz, $CDCl_3$) δ 165.5, 148.1, 138.6, 135.3, 132.4, 128.6, 127.8, 127.8, 123.9, 123.8, 121.2, 119.8, 109.9, 55.8, 21.4.

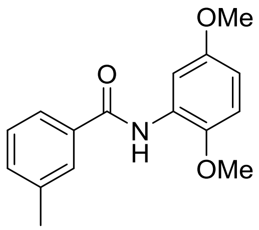

N-(2,5-dimethoxyphenyl)-3-methylbenzamide (3f):

^{1}H NMR (400 MHz, Chloroform-*d*) δ 8.56 (s, 1H), 8.29 (d, *J* = 3.0 Hz, 1H), 7.78 – 7.62 (m, 2H), 7.37 (d, *J* = 7.0 Hz, 2H), 6.83 (d, *J* = 8.9 Hz, 1H), 6.61 (dd, *J* = 8.9, 3.0 Hz, 1H), 3.88 (s, 3H), 3.82 (s, 3H), 2.44 (s, 3H).

^{13}C NMR (101 MHz, $CDCl_3$) δ 165.4, 153.9, 142.3, 138.7, 135.2, 132.5, 128.6, 128.5, 127.8, 123.9, 110.7, 108.8, 105.9, 56.3, 55.8, 21.4.

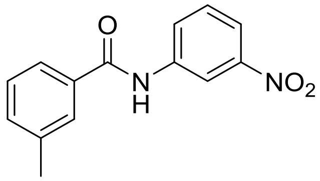

3-methyl-*N*-(3-nitrophenyl)benzamide (3g):

^{1}H NMR (400 MHz, Chloroform-*d*) δ 8.49 (t, *J* = 2.1 Hz, 1H), 8.22 (s, 1H), 8.11 (d, *J* = 8.2 Hz, 1H), 7.98 (d, *J* = 8.2 Hz, 1H), 7.74 – 7.62 (m, 2H), 7.52 (t, *J* = 8.2 Hz, 1H), 7.37 (d, *J* = 5.3 Hz, 2H), 2.42 (s, 3H).

^{13}C NMR (101 MHz, $CDCl_3$) δ 166.2, 148.6, 139.1, 138.9, 133.9, 133.2, 129.9, 128.8, 127.8, 125.9, 124.1, 119.0, 114.9, 21.3.

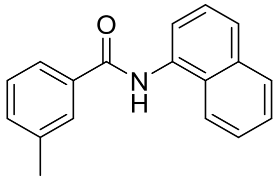

3-methyl-*N*-(naphthalen-1-yl)benzamide (3h):

^{1}H NMR (400 MHz, Chloroform-*d*) δ 8.20 (s, 1H), 8.03 (d, *J* = 7.5 Hz, 1H), 7.90 (dt, *J* = 6.5, 2.9 Hz, 2H), 7.84 – 7.70 (m, 3H), 7.52 (td, *J* = 8.0, 7.3, 4.4 Hz, 3H), 7.41 (d, *J* = 6.0 Hz, 2H), 2.46 (s, 3H).

^{13}C NMR (101 MHz, $CDCl_3$) δ 166.5, 138.6, 134.7, 134.1, 132.6, 132.4, 128.7, 128.6, 128.0, 127.6, 126.2, 126.0, 125.9, 125.7, 124.1, 121.4, 120.9, 21.3.

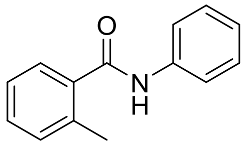

2-methyl-*N*-phenylbenzamide (3i):

^{1}H NMR (400 MHz, Chloroform-*d*) δ 7.65 (d, *J* = 7.9 Hz, 2H), 7.57 (s, 1H), 7.50 (d, *J* = 7.6 Hz, 1H), 7.39 (t, *J* = 8.0 Hz, 3H), 7.29 (t, *J* = 5.3 Hz, 2H), 7.18 (t, *J* = 7.5 Hz, 1H), 2.53 (s, 3H).

^{13}C NMR (101 MHz, $CDCl_3$) δ 168.2, 138.0, 136.4, 131.2, 130.2, 129.1, 126.6, 125.9, 124.5, 119.9, 19.8.

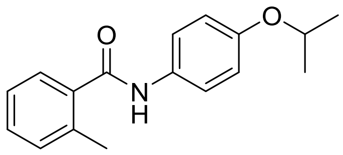

N-(4-isopropoxyphenyl)-2-methylbenzamide (3j):

^{1}H NMR (400 MHz, Chloroform-*d*) δ 7.84 – 7.61 (m, 3H), 7.54 (d, *J* = 8.4 Hz, 2H), 7.44 – 7.32 (m, 2H), 6.92 (d, *J* = 8.4 Hz, 2H), 4.63 – 4.47 (m, 1H), 2.45 (s, 3H), 1.36 (d, *J* = 6.0 Hz, 6H).

^{13}C NMR (101 MHz, $CDCl_3$) δ 166.2, 154.7, 138.4, 134.9, 132.3, 131.1, 128.4, 127.9, 124.1, 122.3, 116.3, 70.2, 22.0, 21.3.

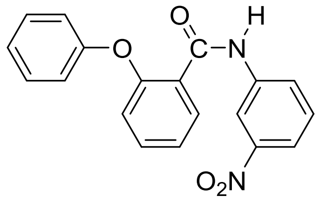

N-(3-nitrophenyl)-2-phenoxybenzamide (3k):

^{1}H NMR (400 MHz, Chloroform-*d*) δ 9.90 (s, 1H), 8.50 (d, *J* = 2.8 Hz, 1H), 8.35 (d, *J* = 7.9 Hz, 1H), 7.98 (dd, *J* = 14.1, 8.4 Hz, 2H), 7.47 (td, *J* = 10.7, 9.1, 5.7 Hz, 4H), 7.29 (d, *J* = 7.5 Hz, 2H), 7.16 (d, *J* = 8.0 Hz, 2H), 6.90 (d, *J* = 8.3 Hz, 1H).

^{13}C NMR (101 MHz, $CDCl_3$) δ 163.1, 155.6, 154.9, 148.6, 139.2, 133.7, 132.6, 130.5, 129.7, 126.0, 125.4, 124.0, 123.0, 119.8, 118.9, 118.2, 115.1.

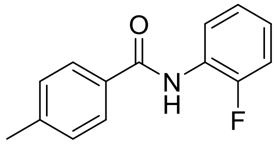

N-(2-fluorophenyl)-4-methylbenzamide (3l):

^{1}H NMR (400 MHz, Chloroform-*d*) δ 8.40 (d, *J* = 15.6 Hz, 1H), 8.24 – 8.12 (m, 1H), 7.60 – 7.46 (m, 3H), 7.31 (t, *J* = 7.6 Hz, 1H), 7.23 – 7.12 (m, 3H), 2.35 (s, 3H).

^{13}C NMR (101 MHz, Chloroform-*d*) δ 161.2, 160.4 (d, *J* = 246.2 Hz), 135.1, 134.4, 133.6 (d, *J* = 9.4 Hz), 132.3 (d, *J* = 2.0 Hz), 129.5, 125.0 (d, *J* = 3.3 Hz), 121.4 (d, *J* = 11.1 Hz), 120.5, 116.1 (d, *J* = 25.1 Hz).

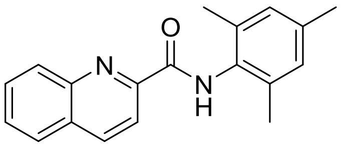

N-mesitylquinoline-2-carboxamide (3m):

^{1}H NMR (400 MHz, Chloroform-*d*) δ 9.67 (s, 1H), 8.41 (q, *J* = 8.6 Hz, 2H), 8.20 (d, *J* = 8.5 Hz, 1H), 7.95 (d, *J* = 8.2 Hz, 1H), 7.83 (t, *J* = 7.8 Hz, 1H), 7.68 (t, *J* = 7.6 Hz, 1H), 6.99 (s, 2H), 2.34 (s, 3H), 2.32 (s, 6H).

^{13}C NMR (101 MHz, $CDCl_3$) δ 162.7, 149.7, 146.5, 137.6, 136.8, 135.2, 131.2, 130.2, 129.8, 129.4, 128.9, 128.0, 127.8, 119.0, 21.0, 18.6.

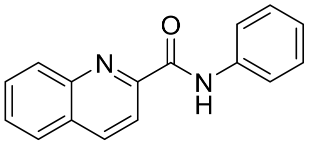

N-phenylquinoline-3-carboxamide (3n):

^{1}H NMR (400 MHz, Chloroform-*d*) δ 10.27 (s, 1H), 8.41 (q, *J* = 8.5 Hz, 2H), 8.22 (d, *J* = 8.5 Hz, 1H), 8.00 – 7.78 (m, 4H), 7.68 (t, *J* = 7.6 Hz, 1H), 7.45 (t, *J* = 7.8 Hz, 2H), 7.21 (t, *J* = 7.4 Hz, 1H).

^{13}C NMR (101 MHz, $CDCl_3$) δ 162.1, 149.6, 146.3, 137.9, 137.8, 130.3, 129.7, 129.4, 129.1, 128.2, 127.8, 124.3, 119.7, 118.7.

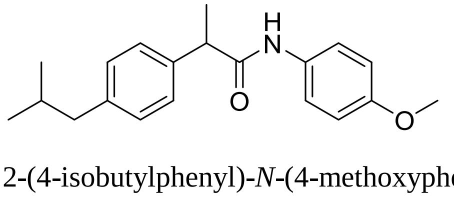

2-(4-isobutylphenyl)-*N*-(4-methoxyphenyl)propenamide (3o):

^{1}H NMR (400 MHz, Chloroform-*d*) δ 7.31 (d, *J* = 8.6 Hz, 2H), 7.26 (d, *J* = 7.2 Hz, 2H), 7.15 (d, *J* = 7.7 Hz, 2H), 6.97 (s, 1H), 6.80 (d, *J* = 8.6 Hz, 2H), 3.76 (s, 3H), 3.67 (q, *J* = 7.2 Hz, 1H), 2.47 (d, *J* = 7.1 Hz, 2H), 1.95 – 1.79 (m, 1H), 1.58 (d, *J* = 7.2 Hz, 3H), 0.91 (d, *J* = 6.6 Hz, 6H).

^{13}C NMR (101 MHz, $CDCl_3$) δ 172.5, 156.3, 140.9, 138.2, 131.1, 129.7, 127.4, 121.6, 114.0, 55.4, 47.4, 45.0, 30.1, 22.3, 18.5.

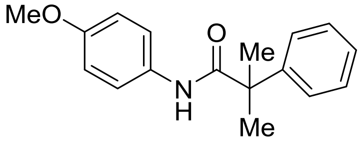

N-(4-methoxyphenyl)-2-methyl-2-phenylpropanamide (3p):

^{1}H NMR (400 MHz, DMSO-d_6) δ 9.00 (s, 1H), 7.47 (d, *J* = 8.5 Hz, 2H), 7.42 – 7.30 (m, 4H), 7.24 (dt, *J* = 6.4, 3.1 Hz, 1H), 6.84 (d, *J* = 8.8 Hz, 2H), 3.70 (s, 3H), 1.55 (s, 6H).

^{13}C NMR (101 MHz, DMSO) δ 174.7, 155.7, 146.5, 132.7, 128.7, 126.7, 126.1, 122.3, 113.9, 55.5, 47.5, 27.3.

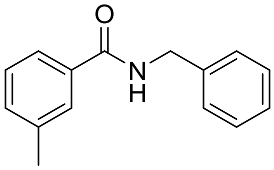

N-benzyl-3-methylbenzamide (3q):

^{1}H NMR (400 MHz, Chloroform-*d*) δ 7.61 (s, 1H), 7.57 (s, 1H), 7.39 – 7.26 (m, 7H), 6.56 (s, 1H), 4.61 (d, *J* = 5.7 Hz, 2H), 2.37 (s, 3H).

^{13}C NMR (101 MHz, $CDCl_3$) δ 167.6, 138.4, 138.3, 134.3, 132.2, 128.7, 128.4, 127.9, 127.7, 127.5, 123.9, 44.1, 21.3.

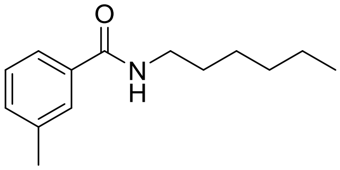

N-hexyl-3-methylbenzamide (3r):

^{1}H NMR (400 MHz, Chloroform-*d*) δ 7.51 (s, 1H), 7.49 – 7.42 (m, 1H), 7.20 (d, *J* = 4.7 Hz, 2H), 6.30 (s, 1H), 3.44 – 3.27 (m, 2H), 2.29 (s, 3H), 1.58 – 1.44 (m, 2H), 1.32 – 1.16 (m, 6H), 0.87 – 0.75 (m, 3H).

^{13}C NMR (101 MHz, $CDCl_3$) δ 167.8, 138.2, 134.8, 131.9, 128.3, 127.7, 123.8, 40.1, 31.5, 29.6, 26.6, 22.5, 21.3, 14.0.

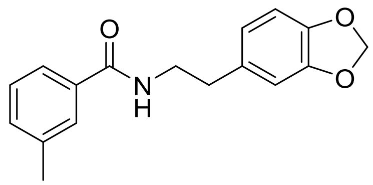

N-(benzo[d][1,3]dioxol-5-yl)-3-methylbenzamide (3s):

^{1}H NMR (400 MHz, Chloroform-*d*) δ 7.54 (s, 1H), 7.49 – 7.42 (m, 1H), 7.28 – 7.24 (m, 2H), 6.81 – 6.60 (m, 3H), 6.26 (s, 1H), 3.64 (q, *J* = 6.6 Hz, 2H), 2.83 (t, *J* = 6.9 Hz, 2H), 2.37 (s, 3H).

^{13}C NMR (101 MHz, $CDCl_3$) δ 167.7, 147.9, 146.2, 138.4, 134.6, 132.6, 132.1, 128.4, 127.6, 123.7, 121.7, 109.1, 108.3, 100.9, 41.3, 35.4, 21.3.

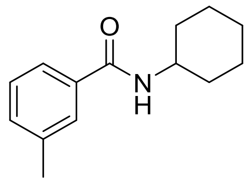

N-cyclohexyl-3-methylbenzamide (3t):

^{1}H NMR (400 MHz, Chloroform-*d*) δ 7.57 (s, 1H), 7.55 – 7.48 (m, 1H), 7.35 – 7.27 (m, 2H), 5.93 (s, 1H), 4.05 – 3.90 (m, 1H), 2.40 (s, 3H), 2.10 – 1.96 (m, 2H), 1.84 – 1.56 (m, 4H), 1.52 – 1.35 (m, 2H), 1.29 – 1.18 (m, 2H).

^{13}C NMR (101 MHz, $CDCl_3$) δ 166.8, 138.2, 135.1, 131.8, 128.3, 127.6, 123.8, 48.6, 33.1, 25.5, 24.9, 21.3.

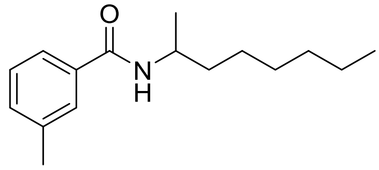

3-methyl-*N*-(octan-2-yl)benzamide (3u):

^{1}H NMR (400 MHz, Chloroform-*d*) δ 7.59 (s, 1H), 7.54 (t, *J* = 4.6 Hz, 1H), 7.26 (d, *J* = 4.6 Hz, 2H), 6.53 (s, 1H), 3.36 (td, *J* = 6.2, 2.0 Hz, 2H), 2.35 (s, 3H), 1.65 – 1.48 (q, *J* = 6.1 Hz, 1H), 1.42 – 1.23 (m, 8H), 0.98 – 0.80 (m, 6H).

^{13}C NMR (101 MHz, $CDCl_3$) δ 167.9, 138.2, 134.9, 131.9, 128.3, 127.7, 123.8, 43.0, 39.4, 31.0, 28.9, 24.3, 23.0, 21.3, 14.0, 10.8.

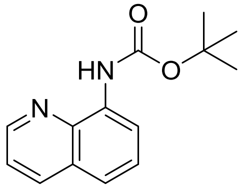

tert-butyl quinolin-8-ylcarbamate (3v):

^{1}H NMR (400 MHz, Chloroform-*d*) δ 9.05 (s, 1H), 8.82 (dd, *J* = 4.1, 2.1 Hz, 1H), 8.44 (d, *J* = 7.6 Hz, 1H), 8.16 (d, *J* = 8.3 Hz, 1H), 7.54 (t, *J* = 8.0 Hz, 1H), 7.45 (d, *J* = 7.8 Hz, 2H), 1.60 (s, 9H).

^{13}C NMR (101 MHz, $CDCl_3$) δ 152.94, 148.00, 138.24, 136.30, 135.18, 128.07, 127.38, 121.56, 120.18, 114.42, 80.45, 28.43.

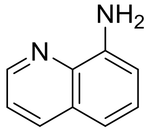

8-aminequinoline (3x):

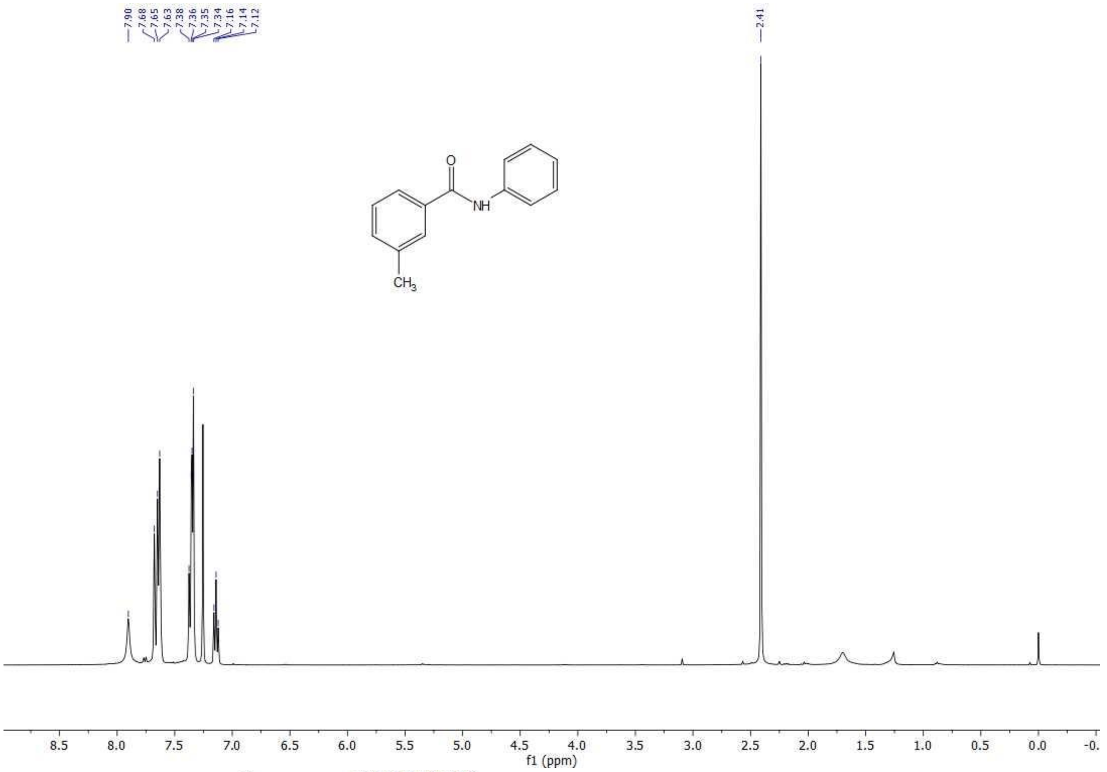

7.90
7.68
7.65
7.63
7.38
7.36
7.35
7.34
7.16
7.14
7.12
2.41
O
NH
CH3
8.5 8.0 7.5 7.0 6.5 6.0 5.5 5.0 4.5 4.0 3.5 3.0 2.5 2.0 1.5 1.0 0.5 0.0
f1 (ppm)

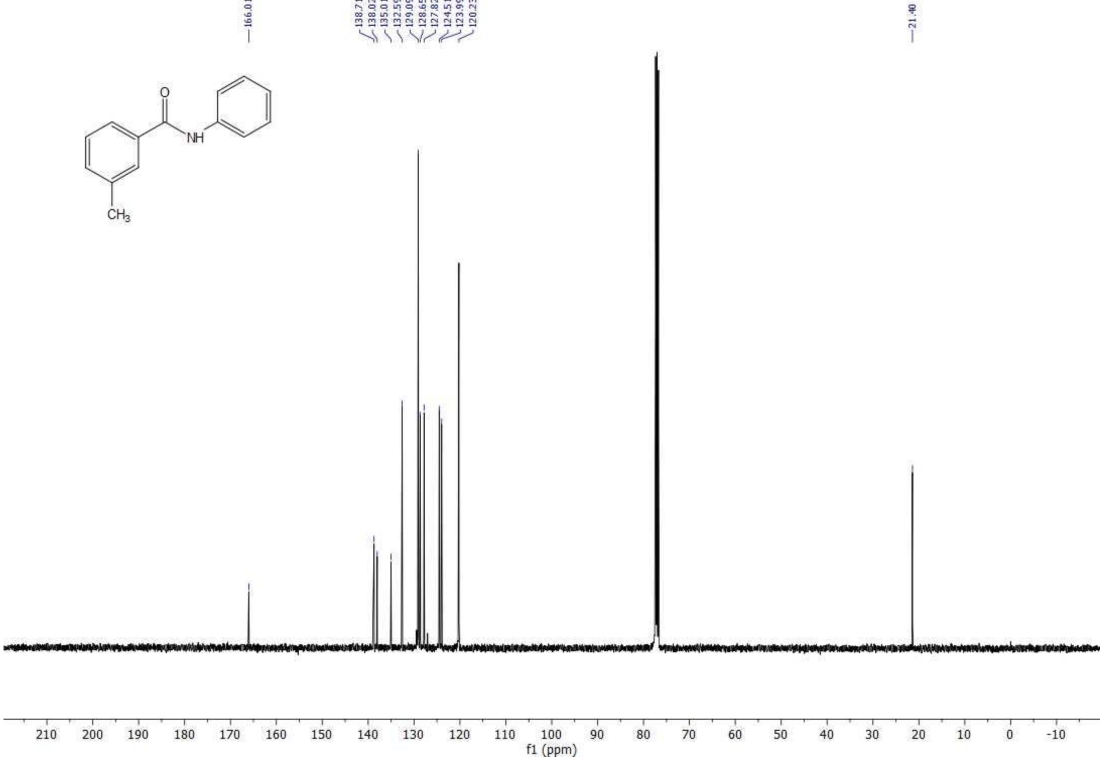

166.01
138.71
138.02
135.01
132.59
129.09
128.65
127.82
124.51
123.99
120.23
21.40
O
NH
CH3
210 200 190 180 170 160 150 140 130 120 110 100 90 80 70 60 50 40 30 20 10 0 -10
f1 (ppm)

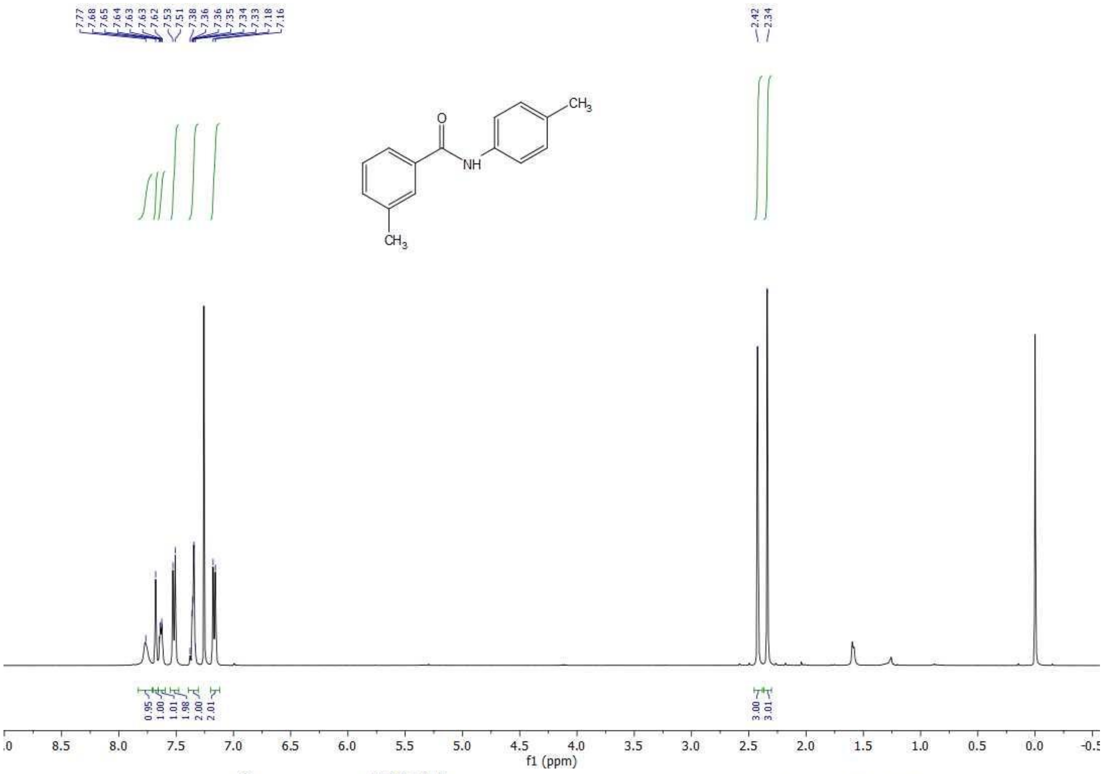
7.77
7.68
7.65
7.64
7.63
7.63
7.62
7.53
7.51
7.38
7.36
7.36
7.35
7.34
7.33
7.18
7.16
2.42
2.34
O
NH
CH3
CH3
0.95
1.00
1.01
1.98
2.00
2.01
3.00
3.01
f1 (ppm)

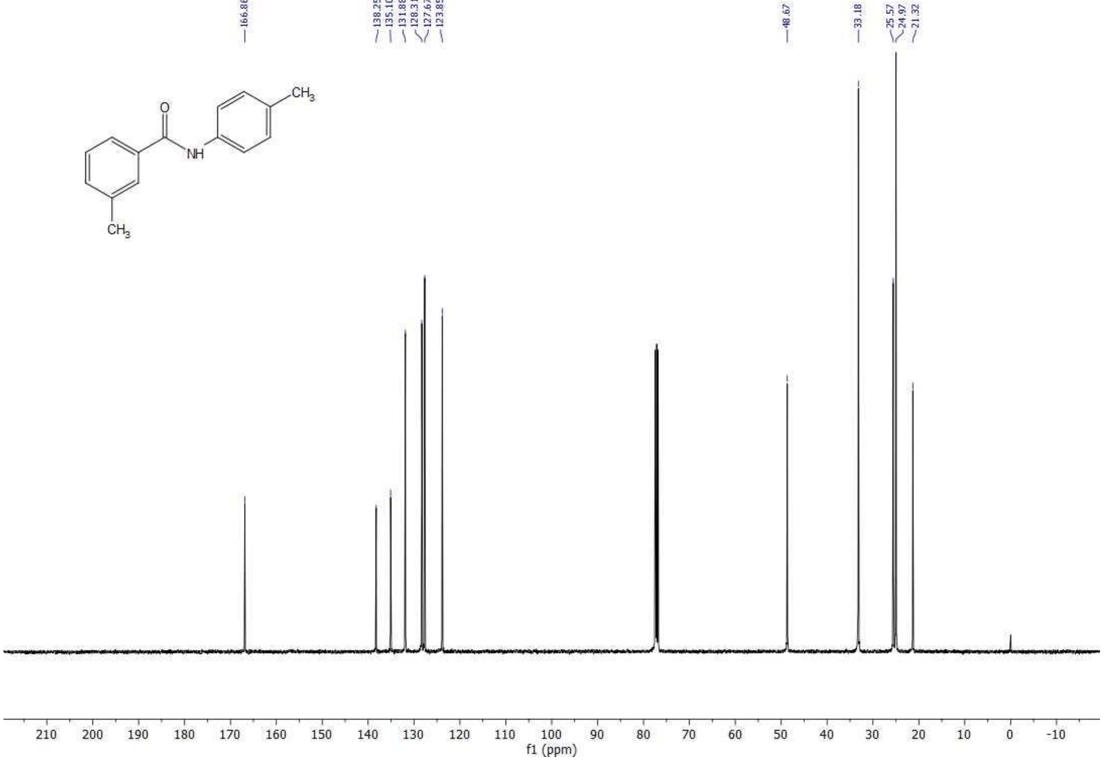
166.86
138.25
135.10
131.88
128.31
127.67
123.85
48.67
33.18
25.57
24.97
21.32
O
NH
CH3
CH3
f1 (ppm)

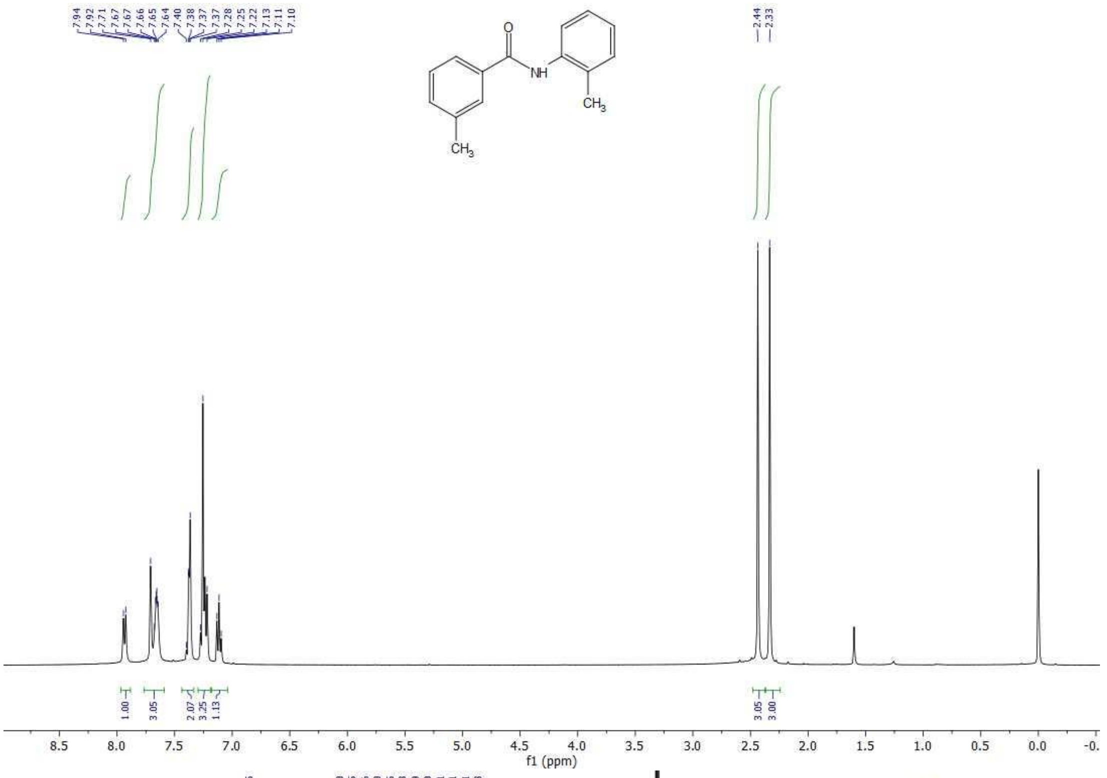
7.94
7.92
7.71
7.67
7.67
7.66
7.65
7.64
7.40
7.38
7.37
7.37
7.28
7.25
7.22
7.13
7.11
7.10
2.44
2.33
O
NH
CH3
CH3
1.00
3.05
2.07
3.25
1.13
3.05
3.00
8.5
8.0
7.5
7.0
6.5
6.0
5.5
5.0
4.5
4.0
3.5
3.0
2.5
2.0
1.5
1.0
0.5
0.0
f1 (ppm)

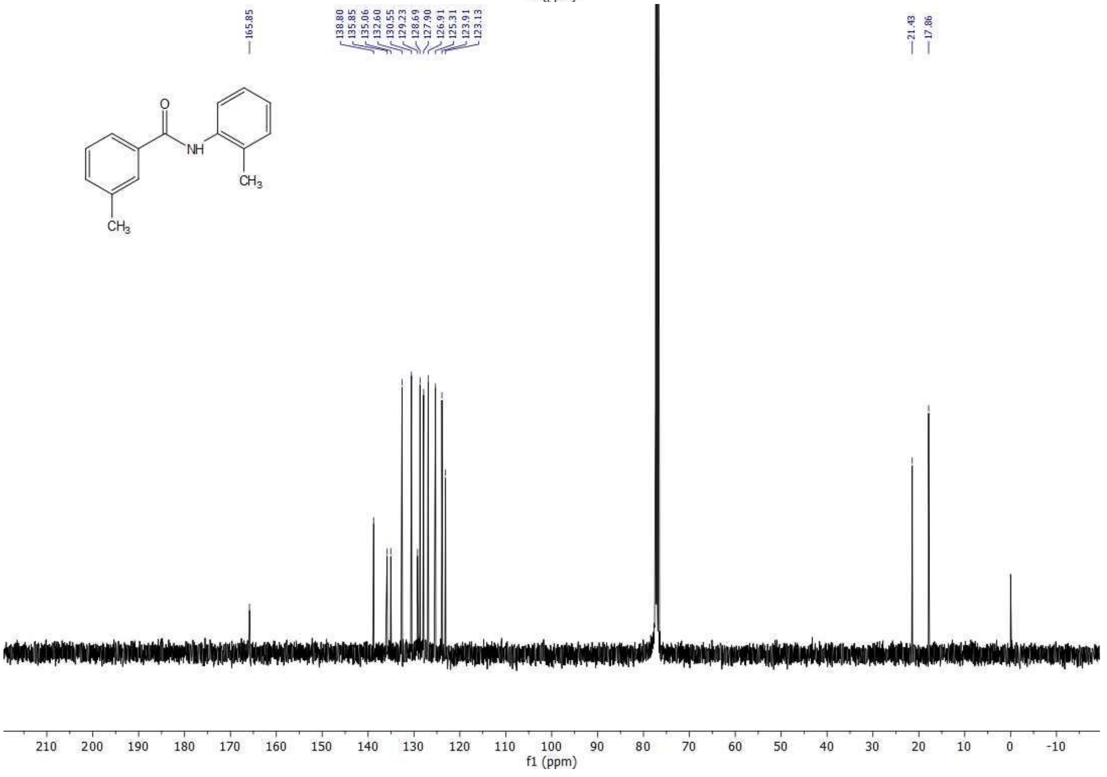
165.85
138.80
135.85
135.06
132.60
130.55
129.23
128.69
127.90
126.91
125.31
123.91
123.13
21.43
17.86
O
NH
CH3
CH3
210
200
190
180
170
160
150
140
130
120
110
100
90
80
70
60
50
40
30
20
10
0
-10
f1 (ppm)

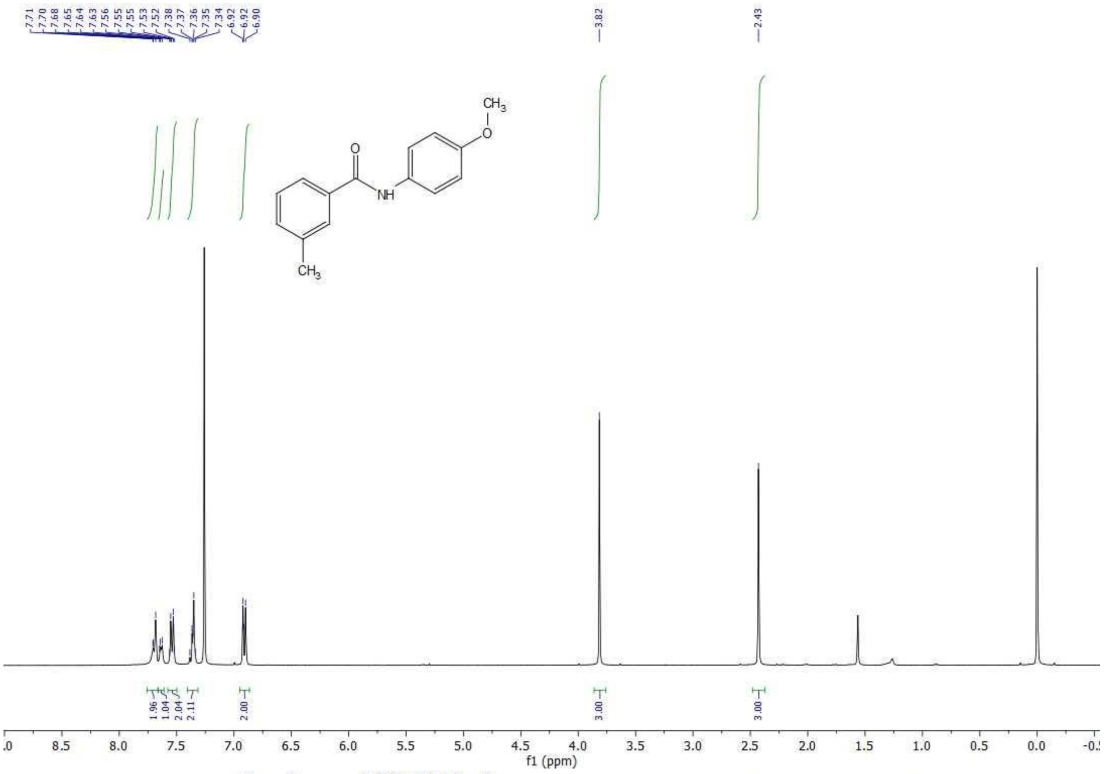

7.71
7.70
7.68
7.65
7.64
7.63
7.56
7.55
7.55
7.53
7.52
7.38
7.37
7.36
7.35
7.34
6.92
6.92
6.90
3.82
2.43
1.96
1.04
2.04
2.11
2.00
3.00
3.00
f1 (ppm)

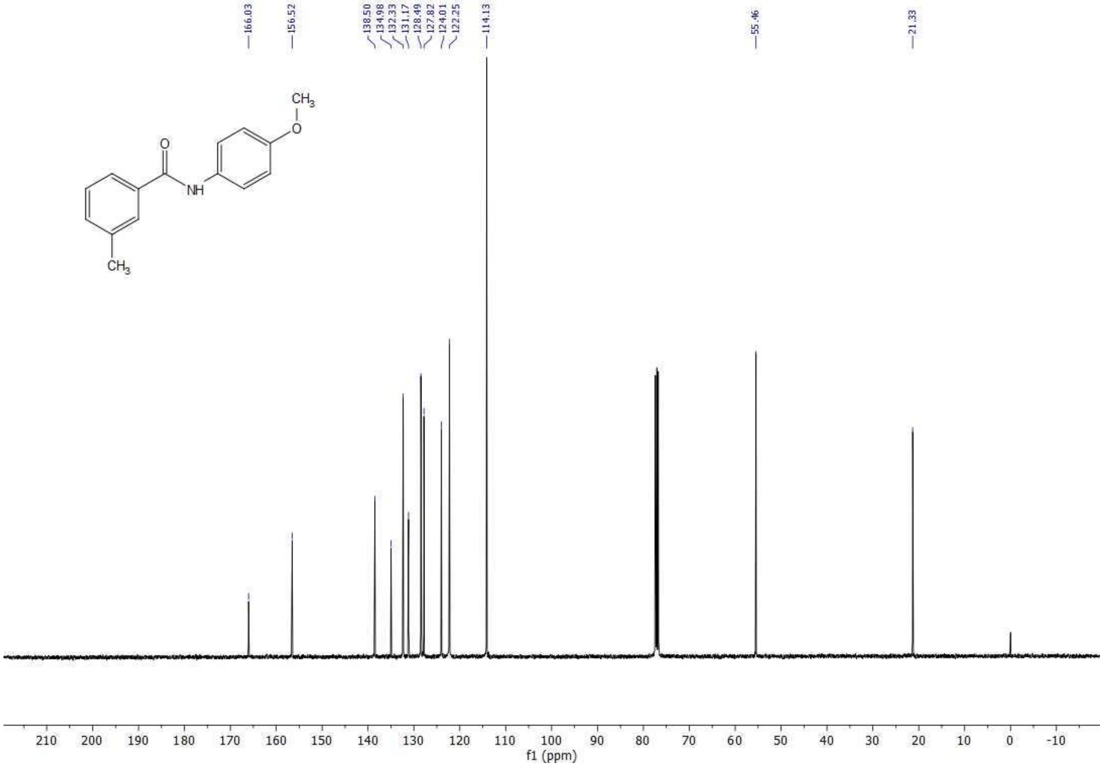

166.03
156.52
138.50
134.98
132.33
131.17
128.49
127.82
124.01
122.25
114.13
55.46
21.33
f1 (ppm)

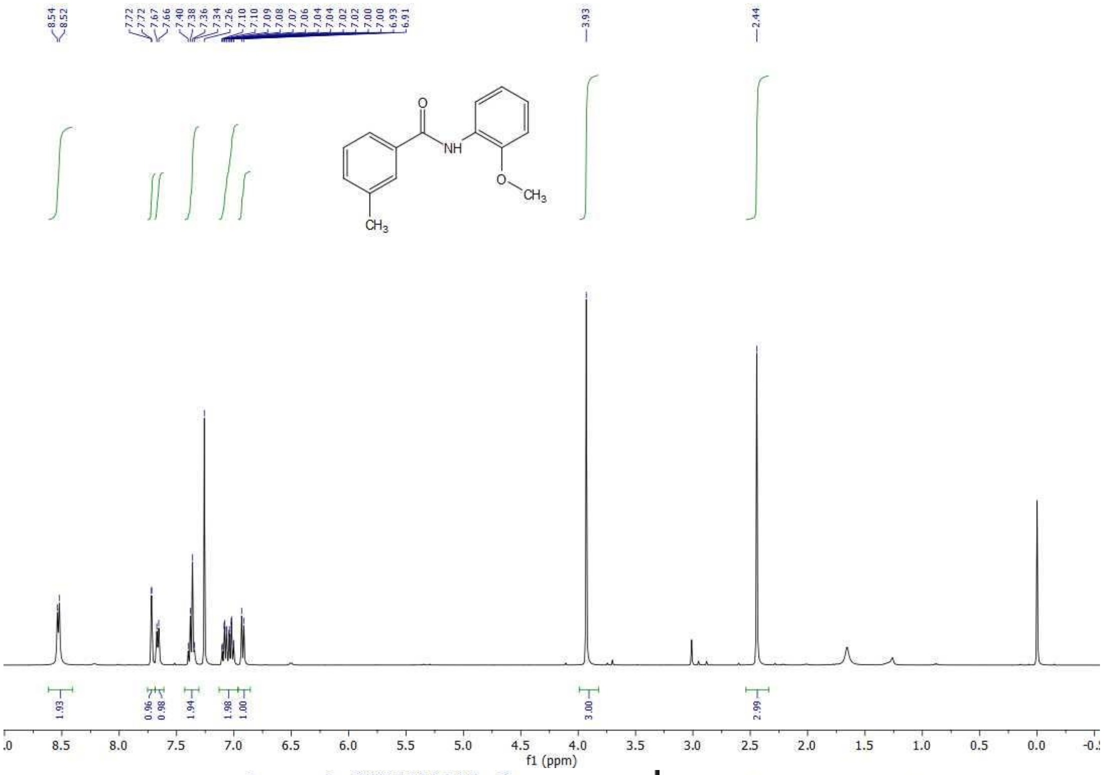

8.54
8.52
7.72
7.72
7.67
7.66
7.40
7.38
7.36
7.34
7.26
7.10
7.09
7.08
7.07
7.06
7.04
7.04
7.02
7.02
7.00
7.00
6.93
6.91
3.93
2.44
O
NH
O
CH3
CH3
1.93
0.96
0.98
1.94
1.98
1.00
3.00
2.99
f1 (ppm)

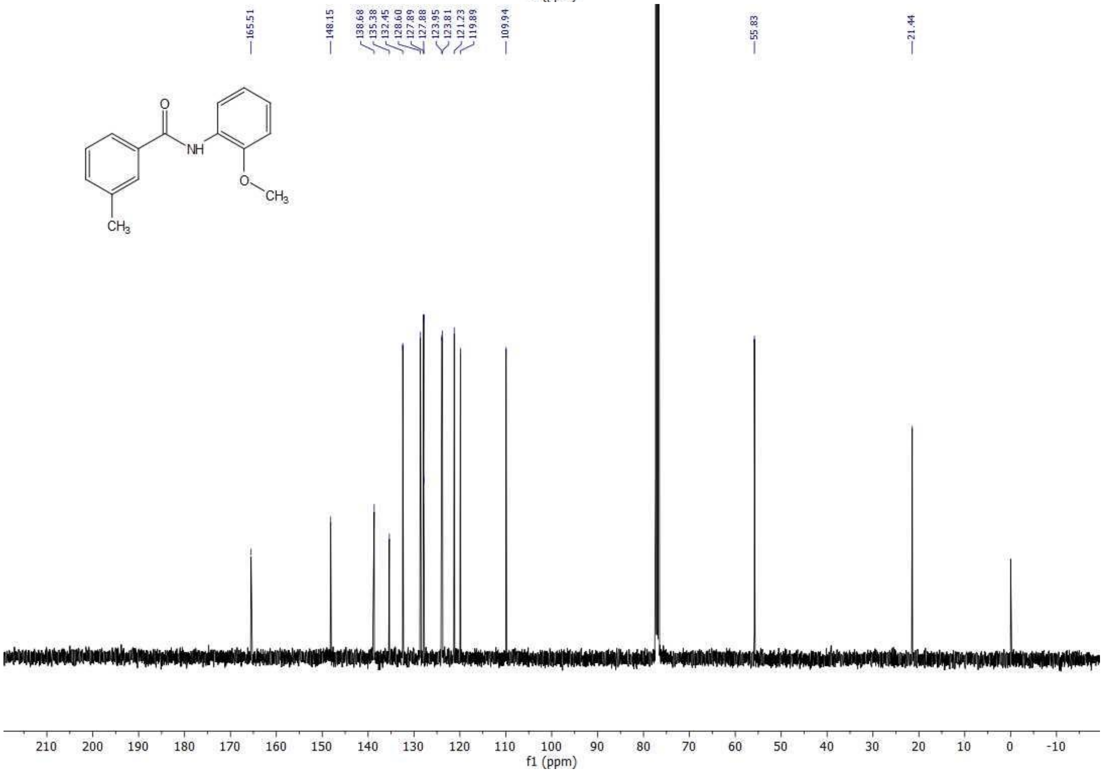

165.51
148.15
138.68
135.38
132.45
128.60
127.89
127.88
123.95
123.81
121.23
119.89
109.94
55.83
21.44
O
NH
O
CH3
CH3
f1 (ppm)

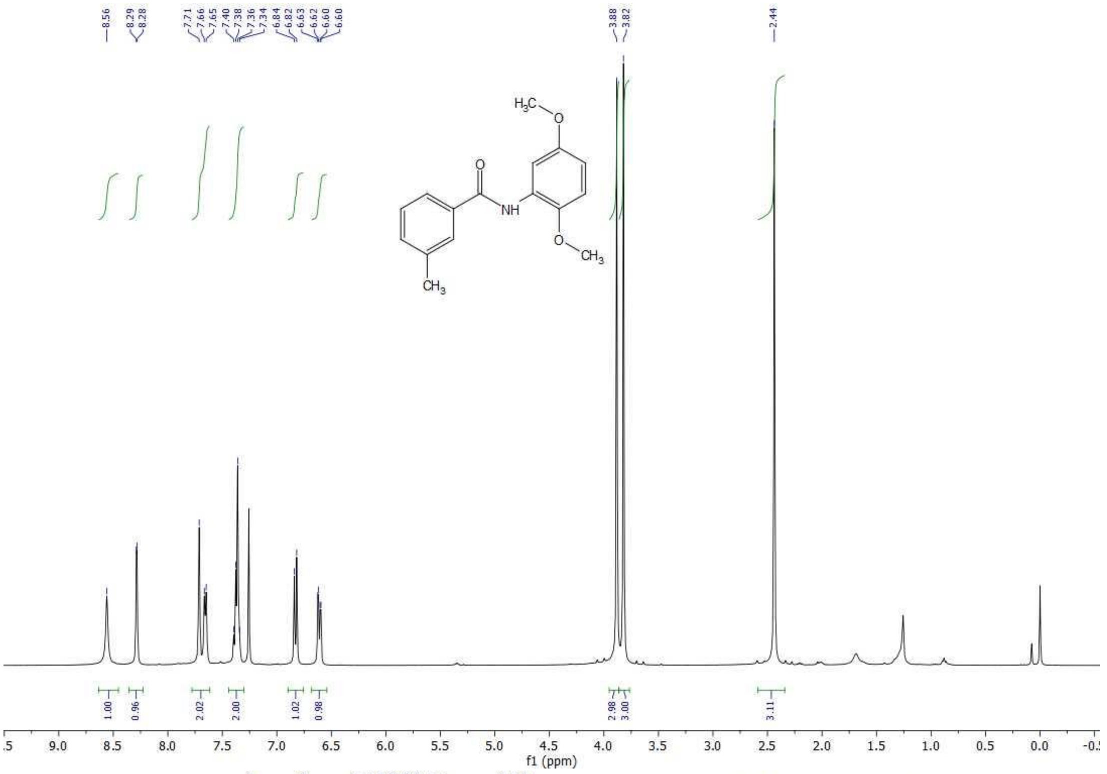
8.56
8.29
8.28
7.71
7.66
7.65
7.40
7.38
7.36
7.34
6.84
6.82
6.63
6.62
6.60
6.60
3.88
3.82
2.44
1.00
0.96
2.02
2.00
1.02
0.98
2.98
3.00
3.11
f1 (ppm)

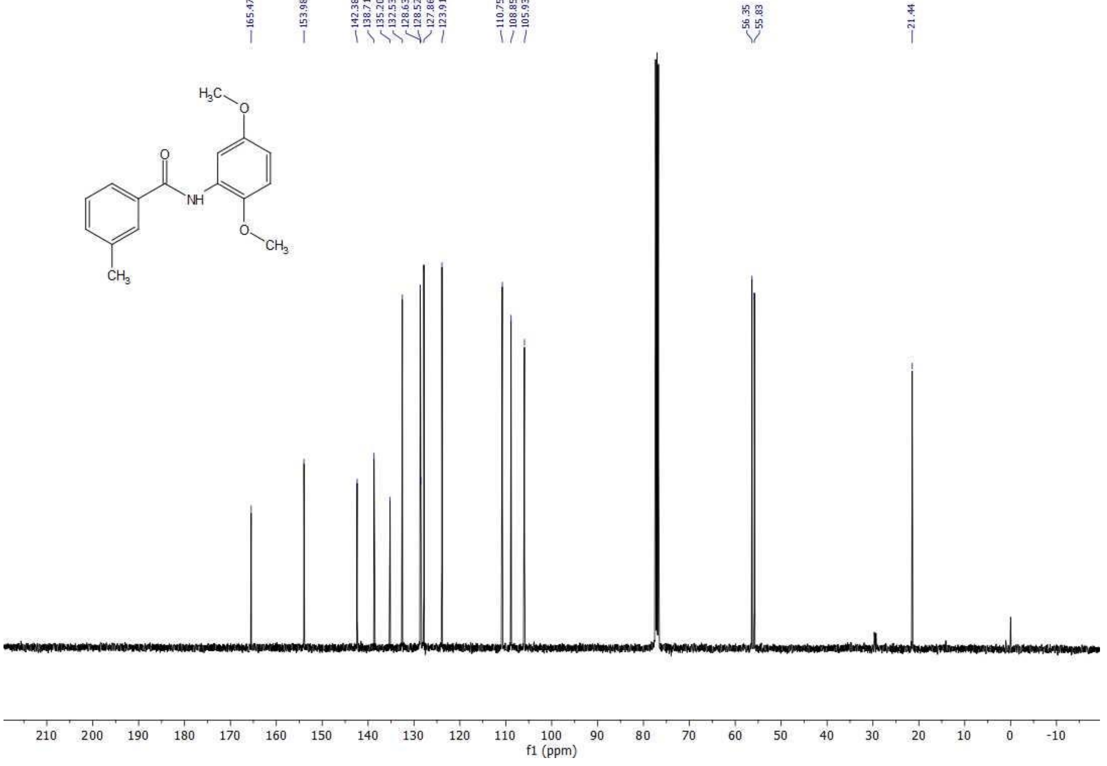
165.47
153.98
142.38
138.71
135.20
132.53
128.63
128.52
127.86
123.91
110.75
108.85
105.93
56.35
55.83
21.44
f1 (ppm)

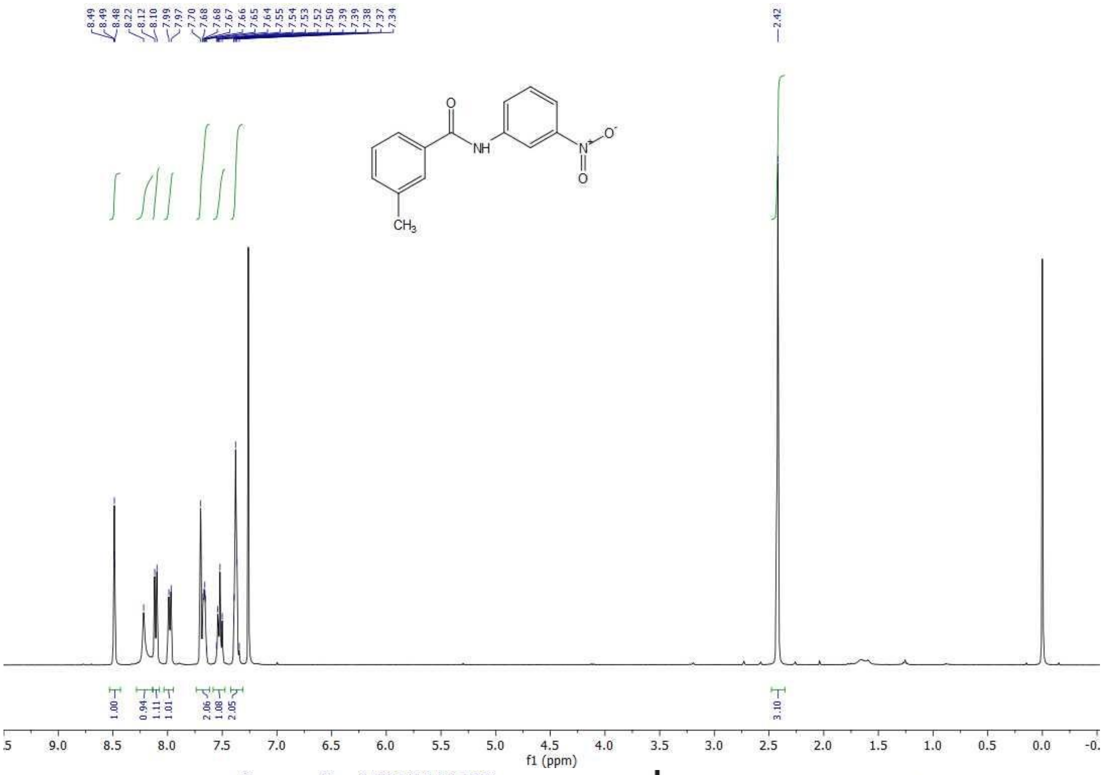
8.49
8.49
8.48
8.22
8.12
8.10
7.99
7.97
7.70
7.68
7.67
7.66
7.65
7.64
7.55
7.54
7.53
7.52
7.50
7.39
7.39
7.38
7.37
7.34
2.42
NH
O
N+
O-
O
CH3
1.00
0.94
1.11
1.01
2.06
1.08
2.05
3.10
f1 (ppm)

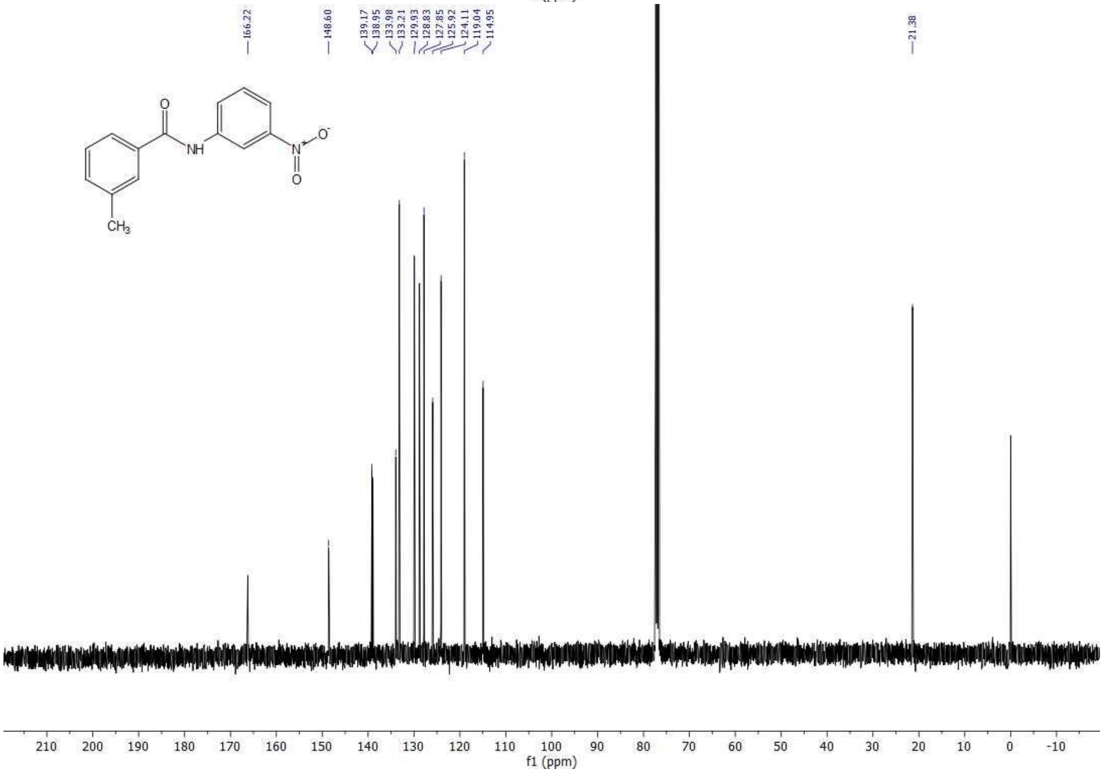
166.22
148.60
139.17
138.95
133.98
133.21
129.93
128.83
127.85
125.92
124.11
119.04
114.95
21.38
O
NH
N+
O-
O
CH3
f1 (ppm)

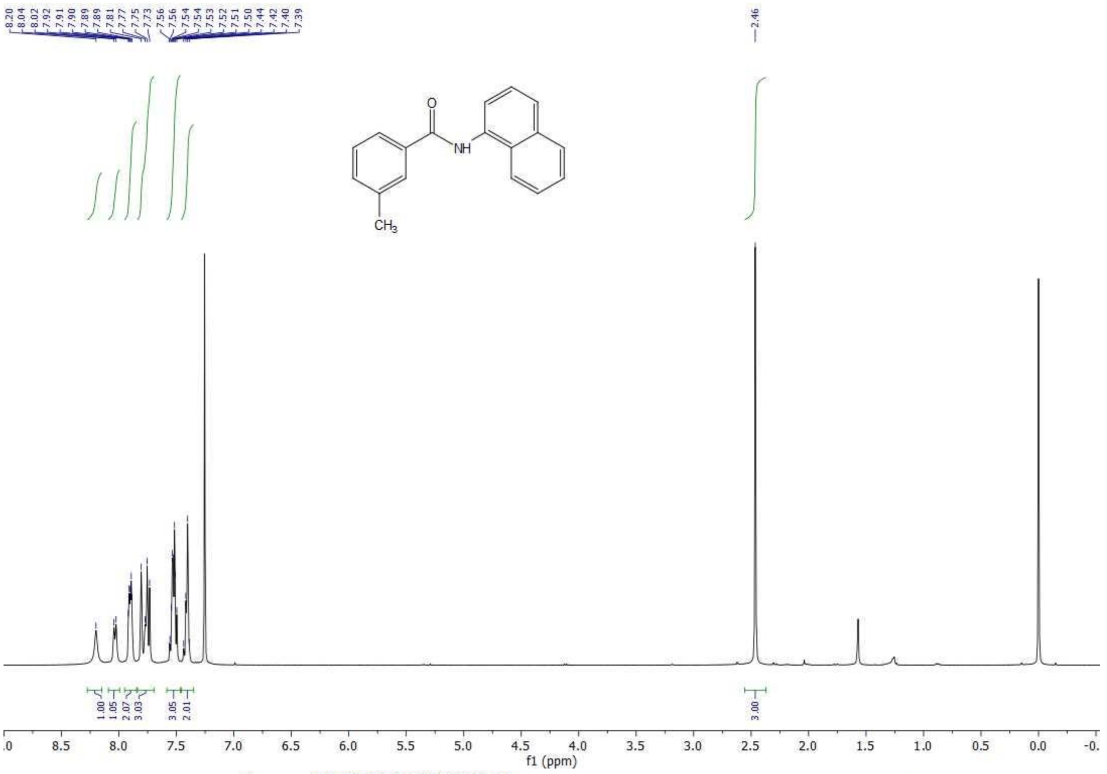

8.20
8.04
8.02
7.92
7.91
7.90
7.89
7.89
7.81
7.77
7.75
7.73
7.56
7.56
7.54
7.54
7.53
7.52
7.51
7.50
7.44
7.42
7.40
7.39
2.46
O
NH
CH3
1.00
1.05
2.07
3.03
3.05
2.01
3.00
f1 (ppm)

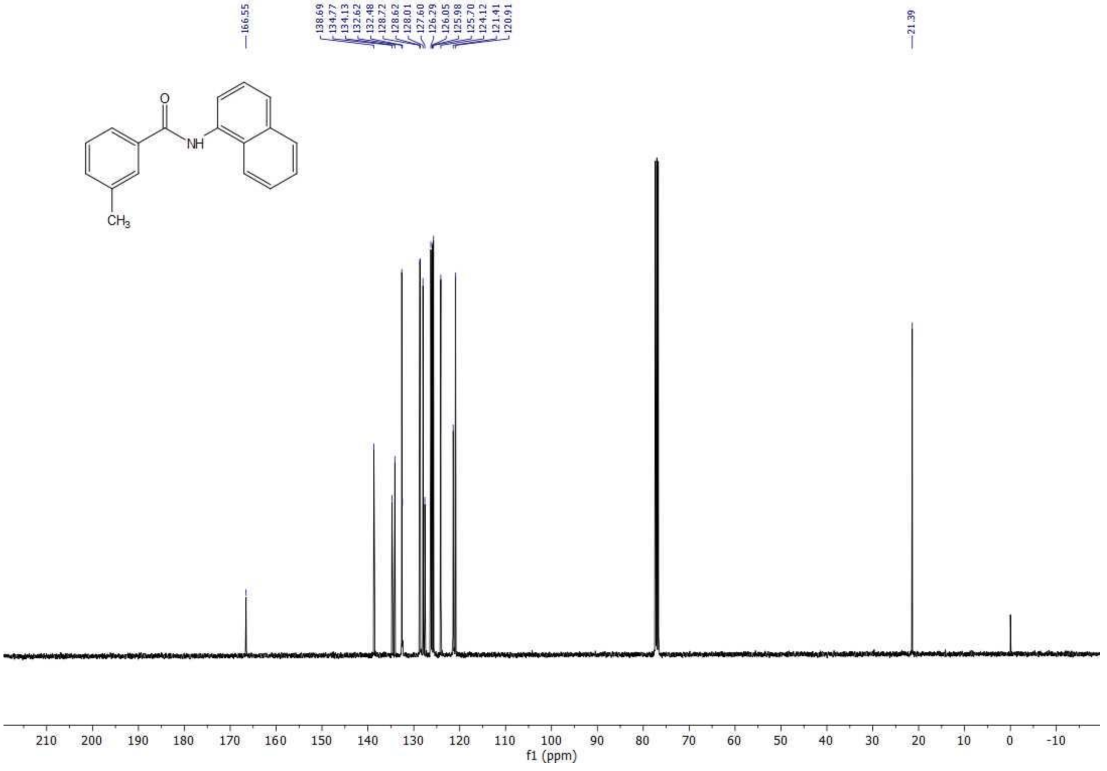

166.55
138.69
134.77
134.13
132.62
132.48
128.72
128.62
128.01
127.60
126.29
126.05
125.98
125.70
124.12
121.41
120.91
21.39
O
NH
CH3
f1 (ppm)

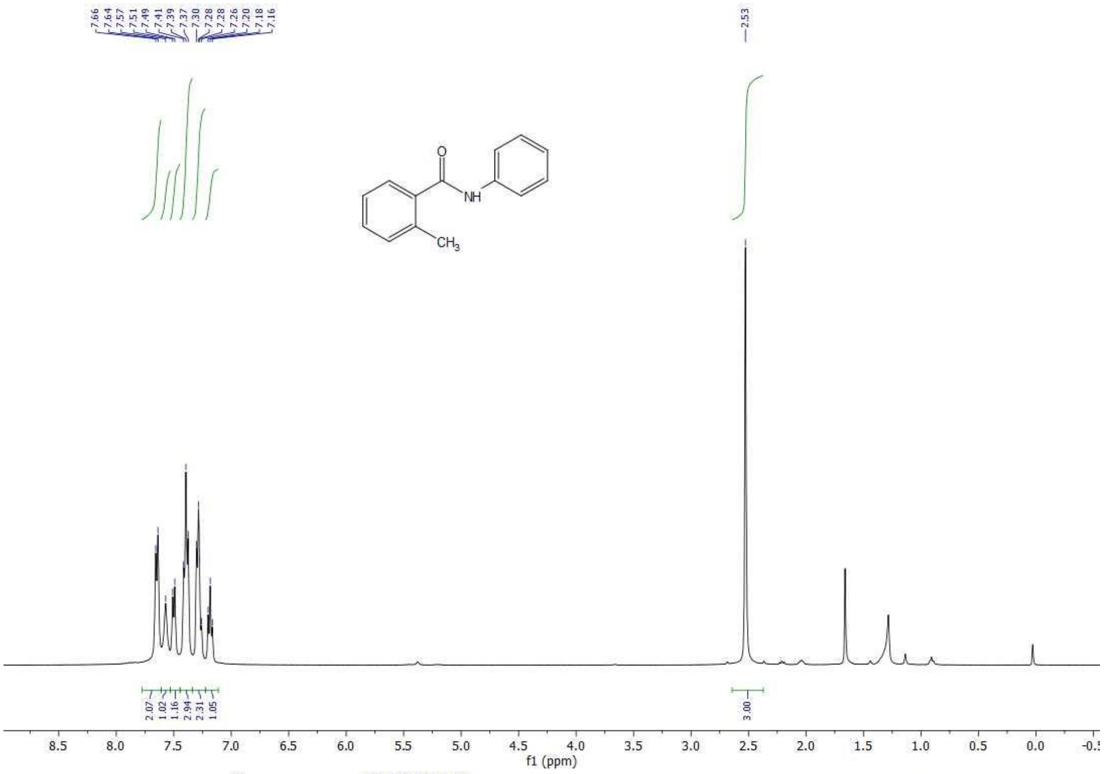

7.66
7.64
7.57
7.51
7.49
7.41
7.39
7.37
7.30
7.28
7.28
7.26
7.20
7.18
7.16
2.53
O
NH
CH3
2.07
1.02
1.16
2.94
2.31
1.05
3.00
f1 (ppm)

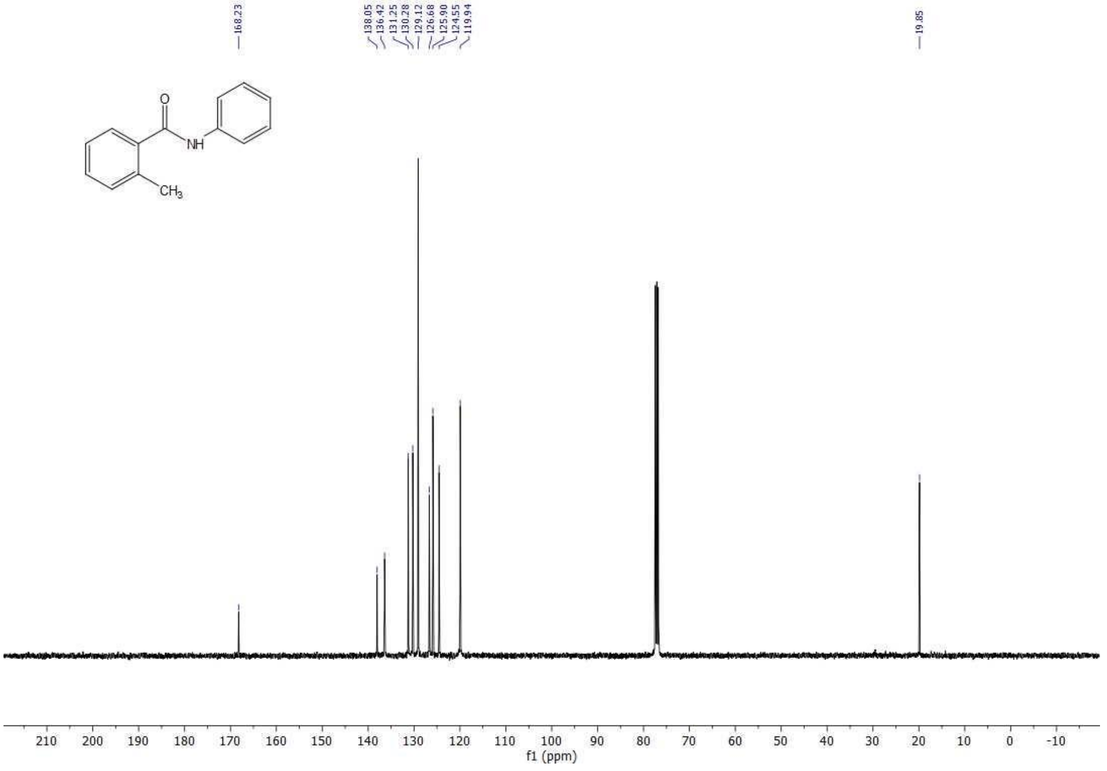

168.23
138.05
136.42
131.25
130.28
129.12
126.68
125.90
124.55
119.94
19.85
O
NH
CH3
f1 (ppm)

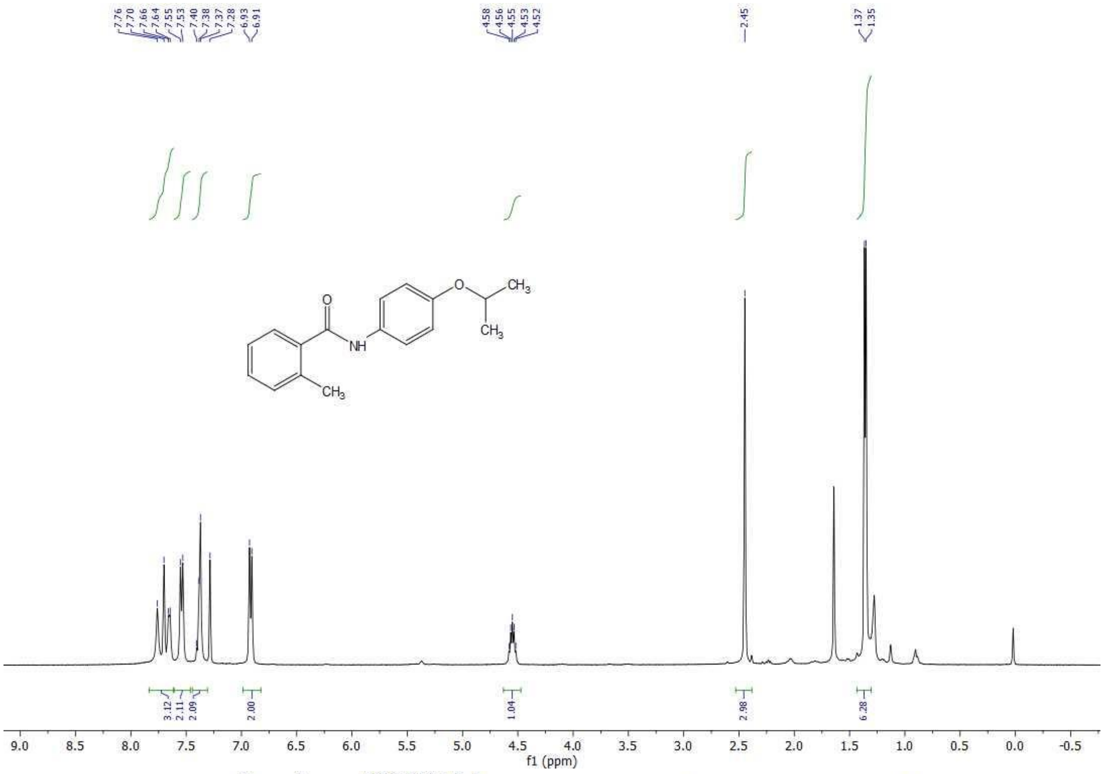

7.76
7.70
7.66
7.64
7.55
7.53
7.40
7.38
7.37
7.28
6.93
6.91
4.58
4.56
4.55
4.53
4.52
2.45
1.37
1.35
3.12
2.11
2.09
2.00
1.04
2.98
6.28
f1 (ppm)

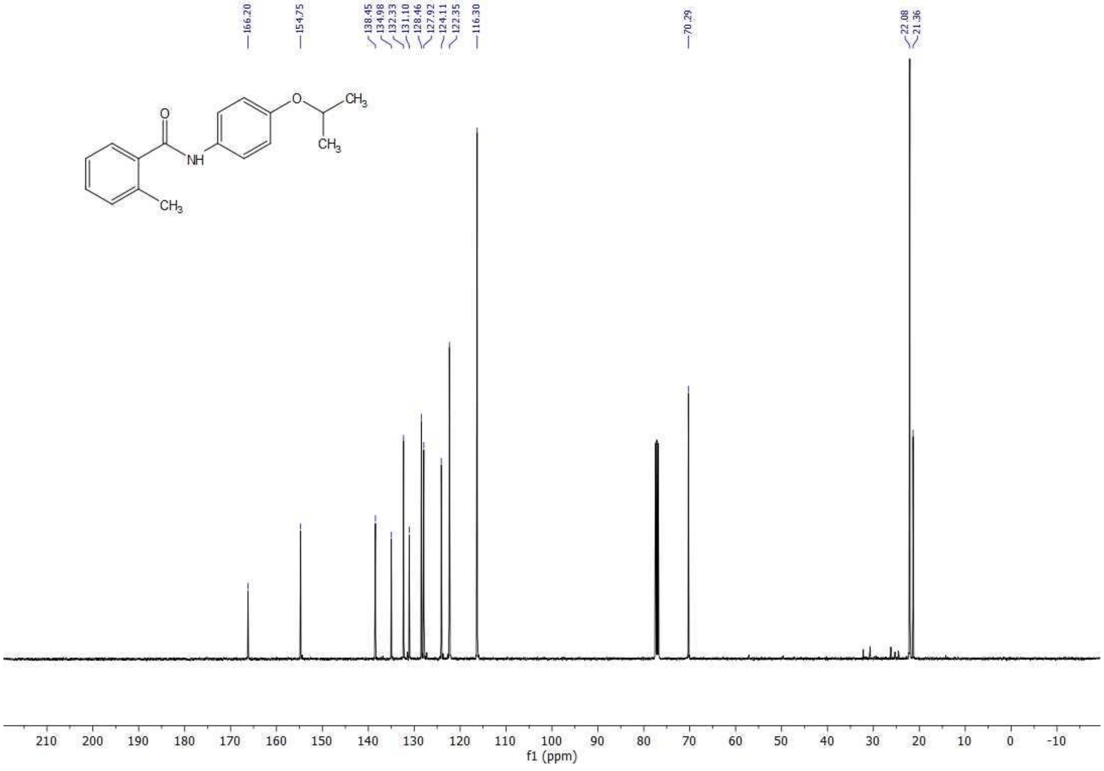

166.20
154.75
138.45
134.98
132.33
131.10
128.46
127.92
124.11
122.35
116.30
70.29
22.08
21.36
f1 (ppm)

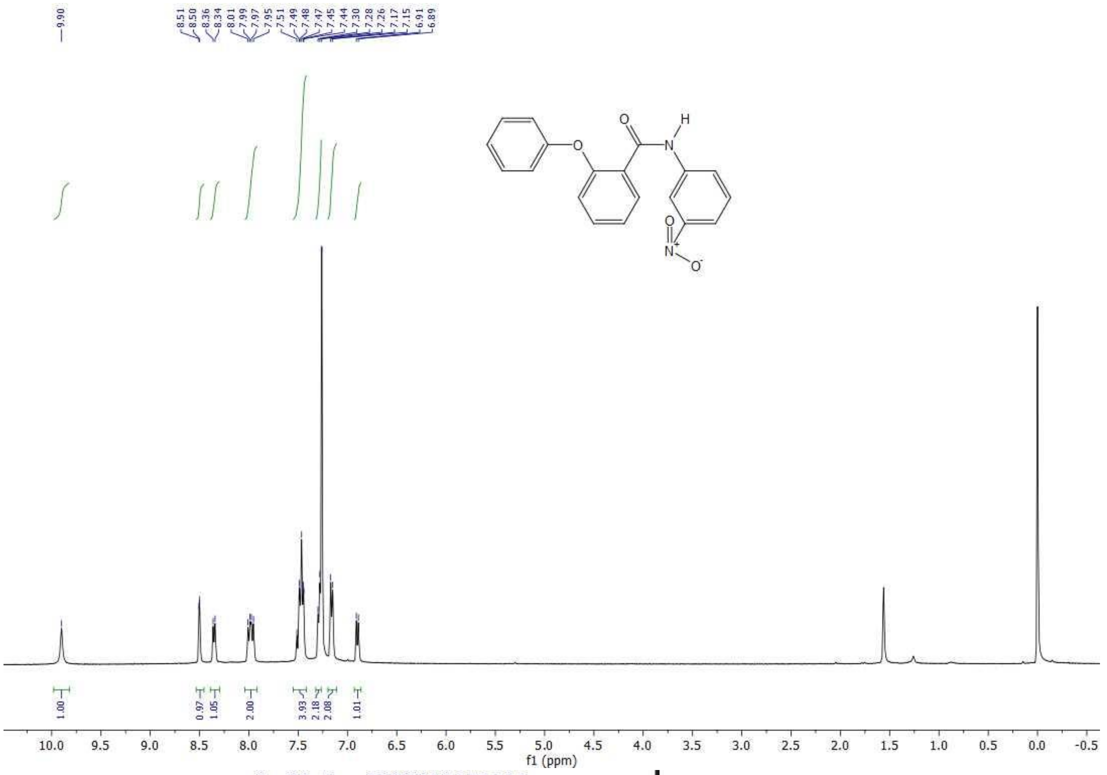

9.90
8.51
8.50
8.36
8.34
8.01
7.99
7.97
7.95
7.51
7.49
7.48
7.47
7.45
7.44
7.30
7.28
7.26
7.17
7.15
6.91
6.89
O
H
N
O
N
O
1.00
0.97
1.05
2.00
3.93
2.18
2.08
1.01
10.0 9.5 9.0 8.5 8.0 7.5 7.0 6.5 6.0 5.5 5.0 4.5 4.0 3.5 3.0 2.5 2.0 1.5 1.0 0.5 0.0 -0.5
f1 (ppm)

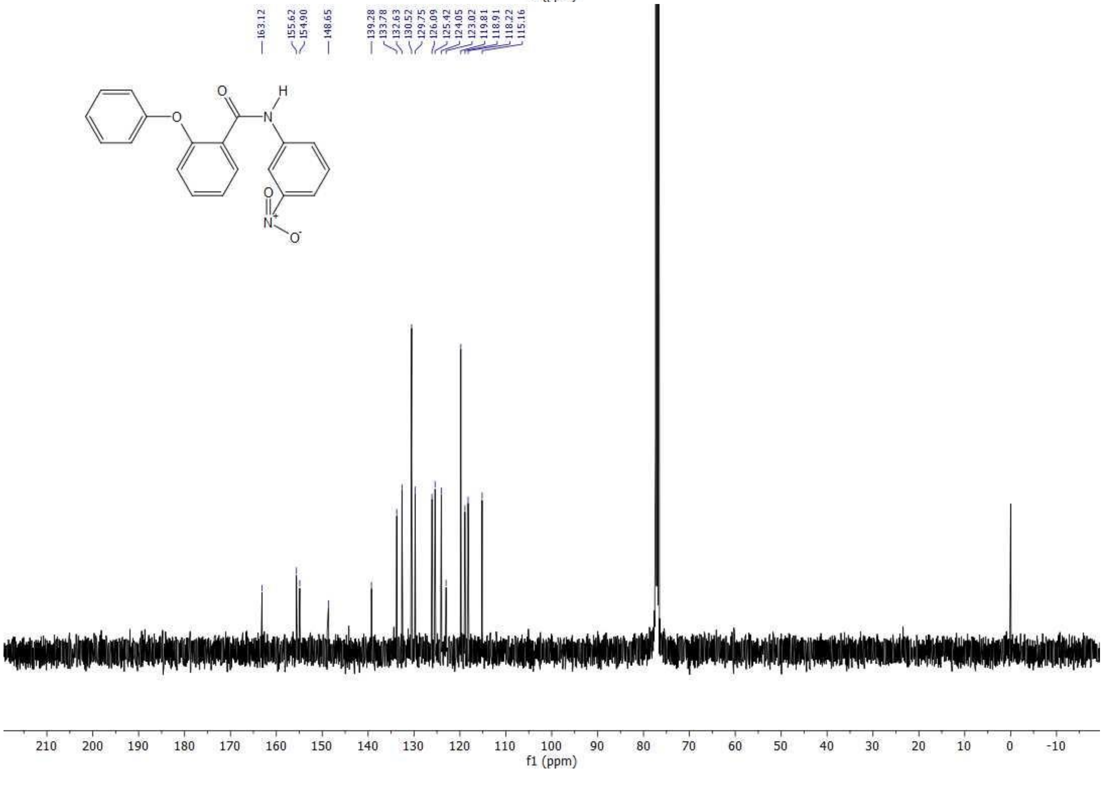

163.12
155.62
154.90
148.65
139.28
133.78
132.63
130.52
129.75
126.09
125.42
124.05
123.02
119.81
118.91
118.22
115.16
O
H
N
O
N
O
210 200 190 180 170 160 150 140 130 120 110 100 90 80 70 60 50 40 30 20 10 0 -10
f1 (ppm)

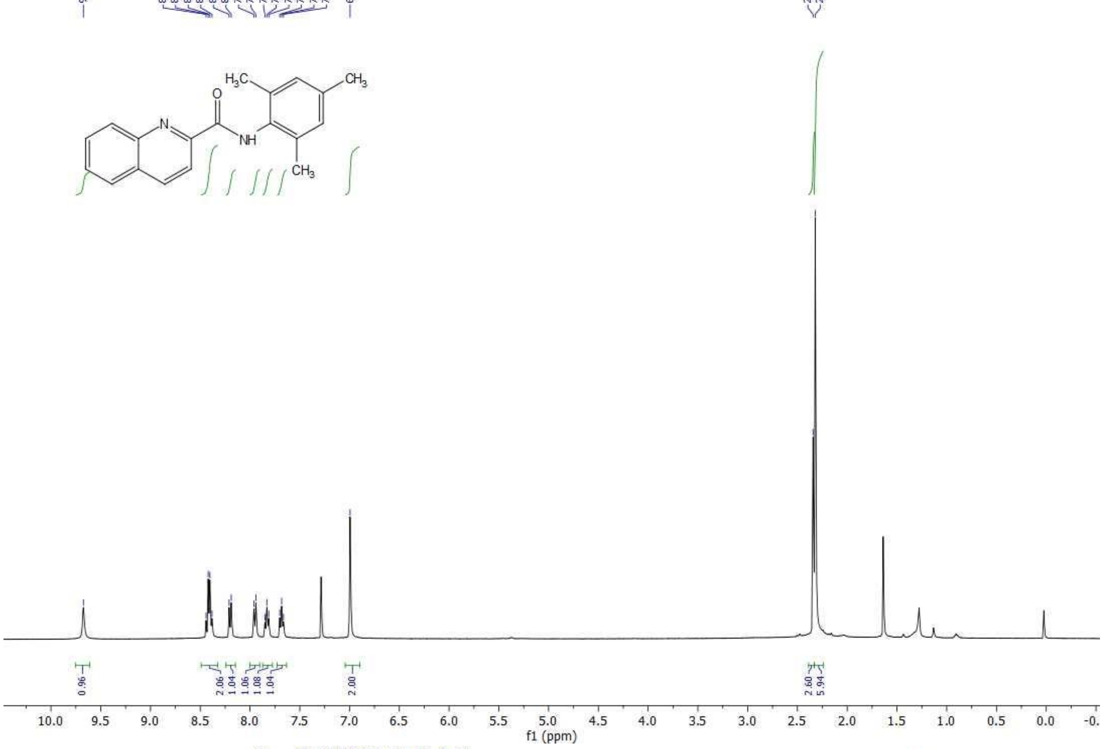
9.67
8.44 8.42 8.40 8.38 8.21 8.19 7.96 7.94 7.85 7.83 7.81 7.70 7.68 7.66
6.99
2.34 2.32
0.96 2.06 1.04 1.06 1.08 1.04 2.00 2.60 5.94
10.0 9.5 9.0 8.5 8.0 7.5 7.0 6.5 6.0 5.5 5.0 4.5 4.0 3.5 3.0 2.5 2.0 1.5 1.0 0.5 0.0 -0.
f1 (ppm)

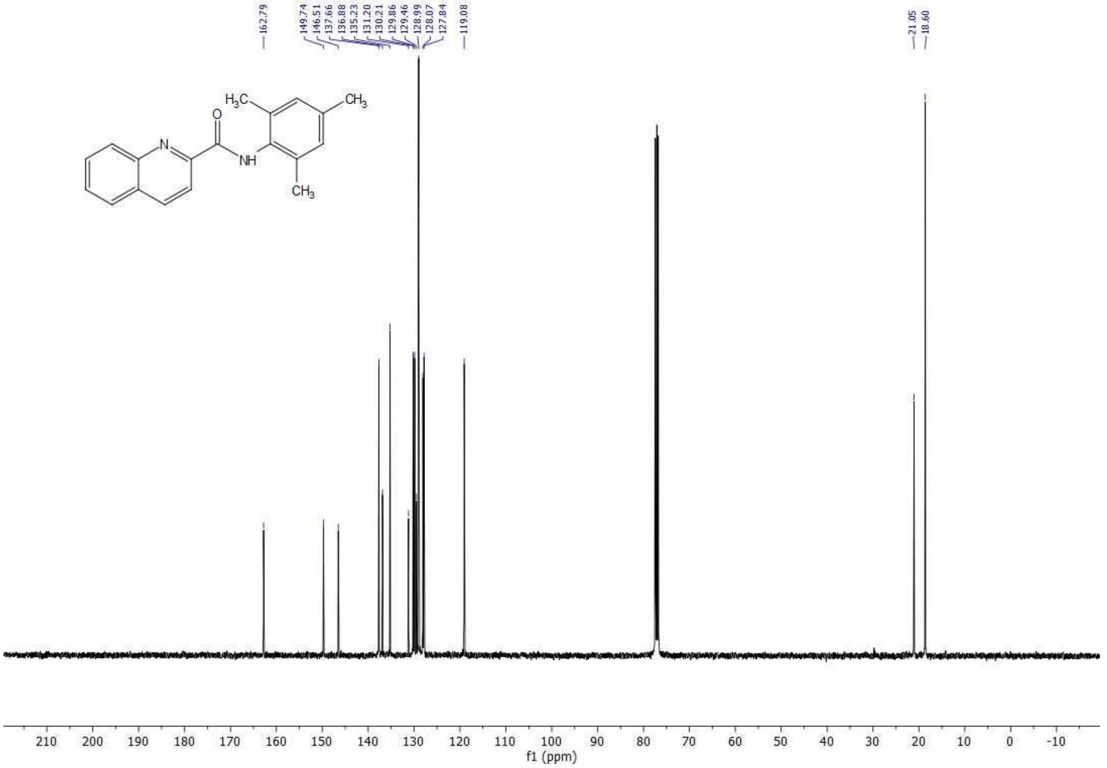
162.79
149.74 146.51 137.66 136.88 135.23 131.20 130.21 129.86 129.46 128.99 128.07 127.84
119.08
21.05 18.60
210 200 190 180 170 160 150 140 130 120 110 100 90 80 70 60 50 40 30 20 10 0 -10
f1 (ppm)

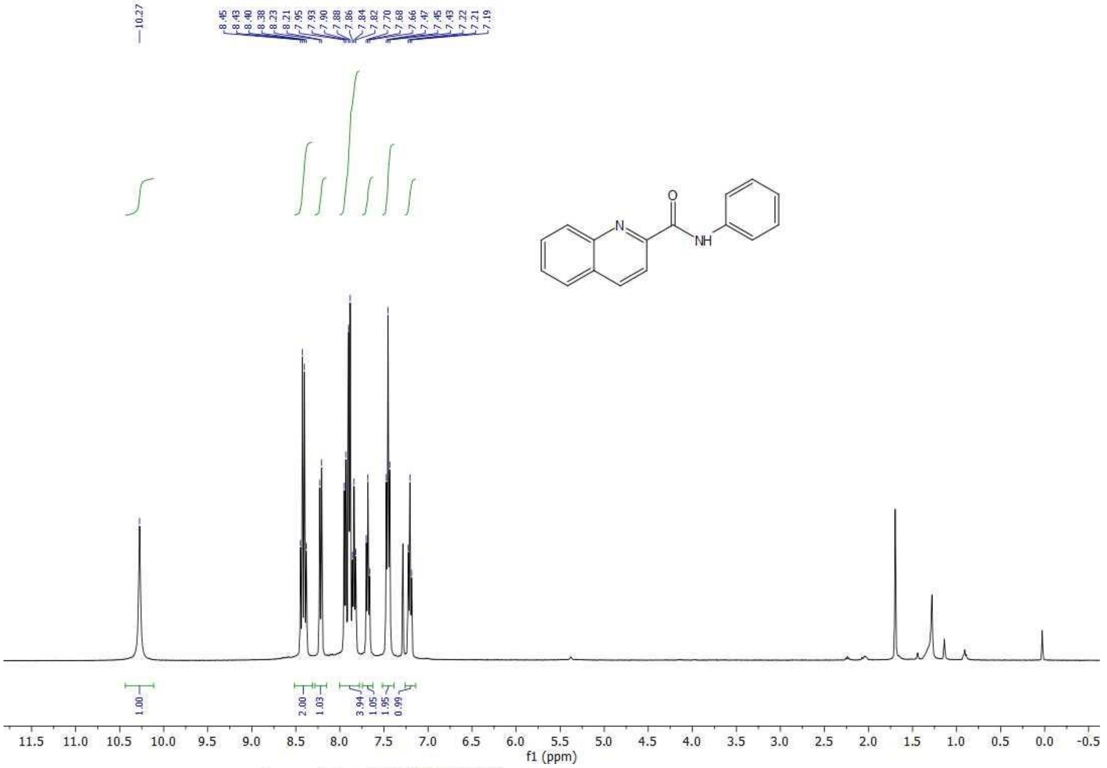

10.27
8.45 8.43 8.40 8.38 8.23 8.21 7.95 7.93 7.90 7.88 7.86 7.84 7.82 7.70 7.68 7.66 7.47 7.45 7.43 7.22 7.21 7.19
O
N
NH
1.00
2.00
1.03
3.94
1.05
1.95
0.99
11.5 11.0 10.5 10.0 9.5 9.0 8.5 8.0 7.5 7.0 6.5 6.0 5.5 5.0 4.5 4.0 3.5 3.0 2.5 2.0 1.5 1.0 0.5 0.0 -0.5
f1 (ppm)

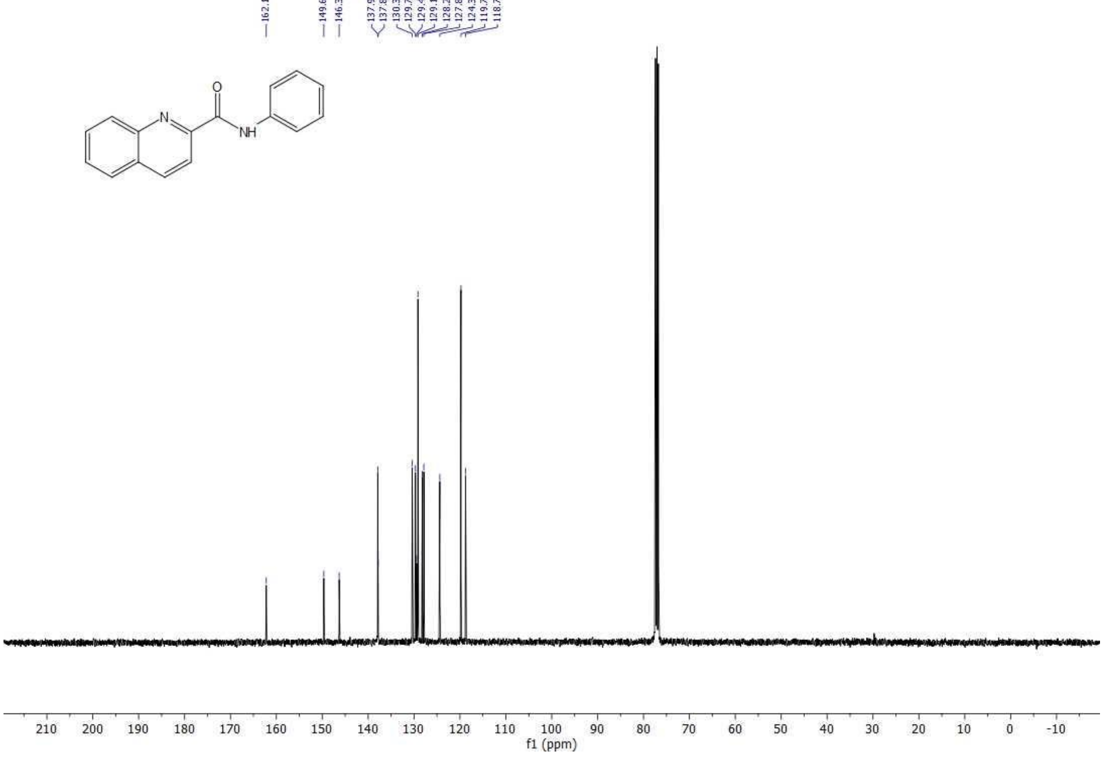

162.19
149.66
146.31
137.92 137.81 130.38 129.70 129.45 129.16 128.21 127.87 124.39 119.78 118.78
O
N
NH
210 200 190 180 170 160 150 140 130 120 110 100 90 80 70 60 50 40 30 20 10 0 -10
f1 (ppm)

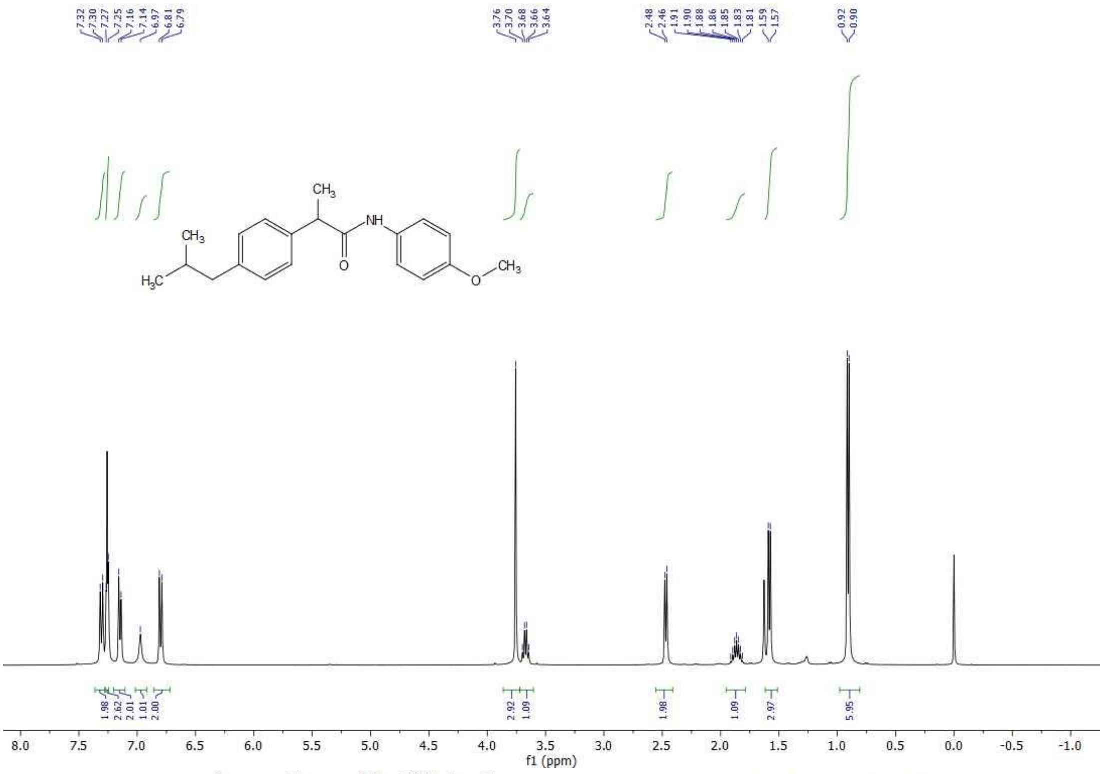
7.32
7.30
7.27
7.25
7.16
7.14
6.97
6.81
6.79
3.76
3.70
3.68
3.66
3.64
2.48
2.46
1.91
1.90
1.88
1.86
1.85
1.83
1.81
1.59
1.57
0.92
0.90
CH3
NH
CH3
H3C
O
O
CH3
1.98
2.62
2.01
1.01
2.00
2.92
1.09
1.98
1.09
2.97
5.95
f1 (ppm)

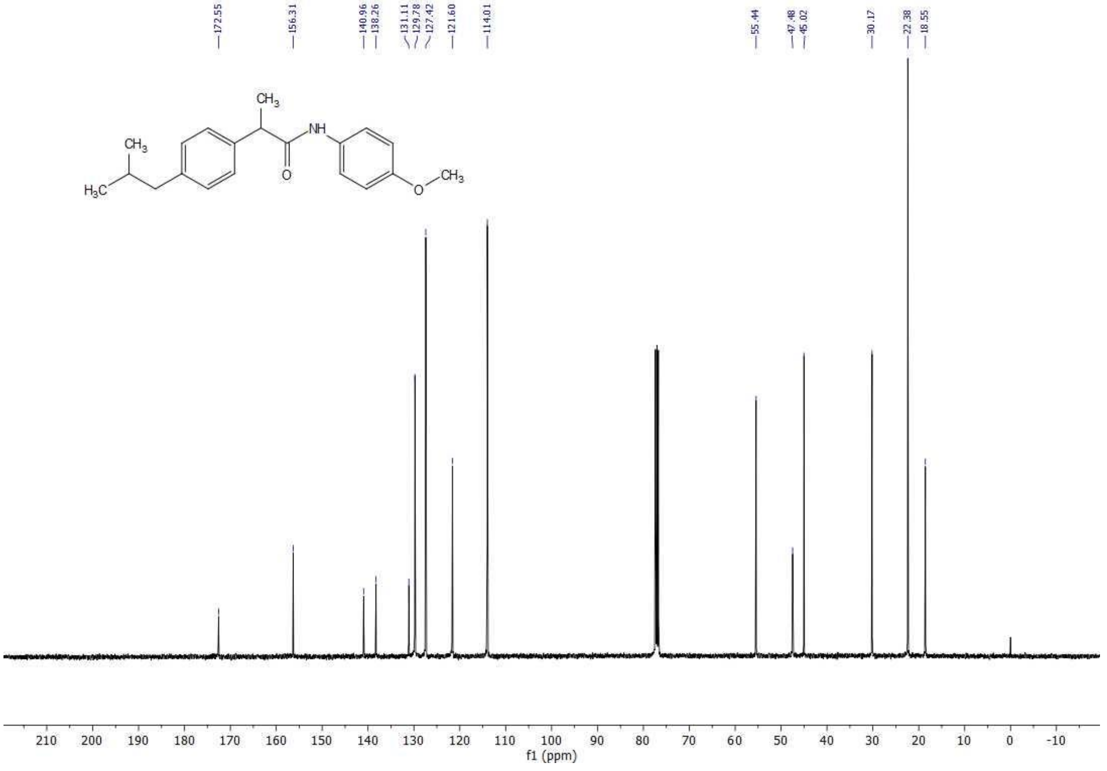
172.55
156.31
140.96
138.26
131.11
129.78
127.42
121.60
114.01
55.44
47.48
45.02
30.17
22.38
18.55
CH3
NH
CH3
H3C
O
O
CH3
f1 (ppm)

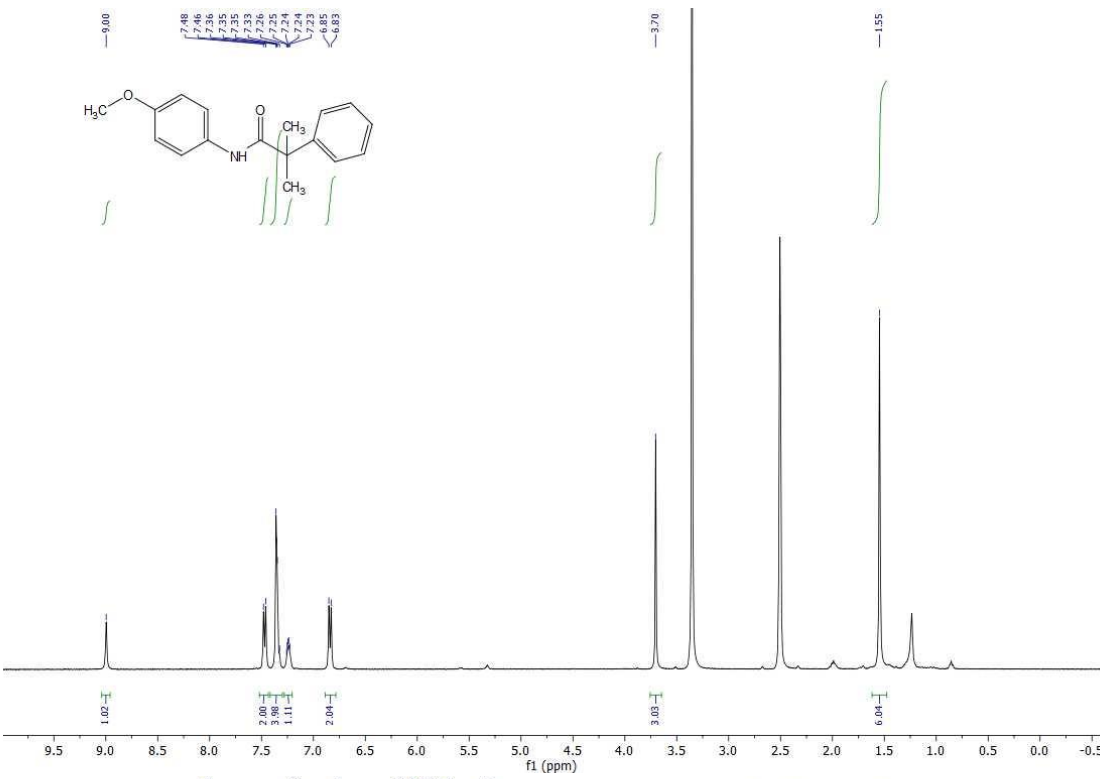

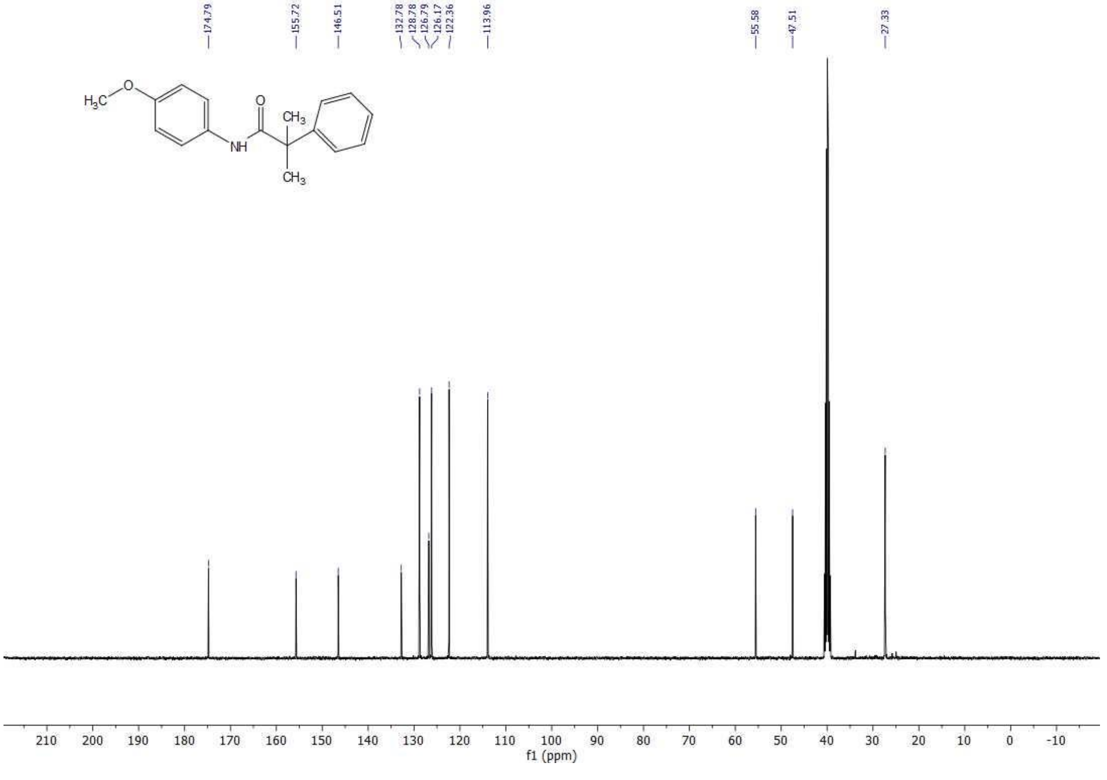

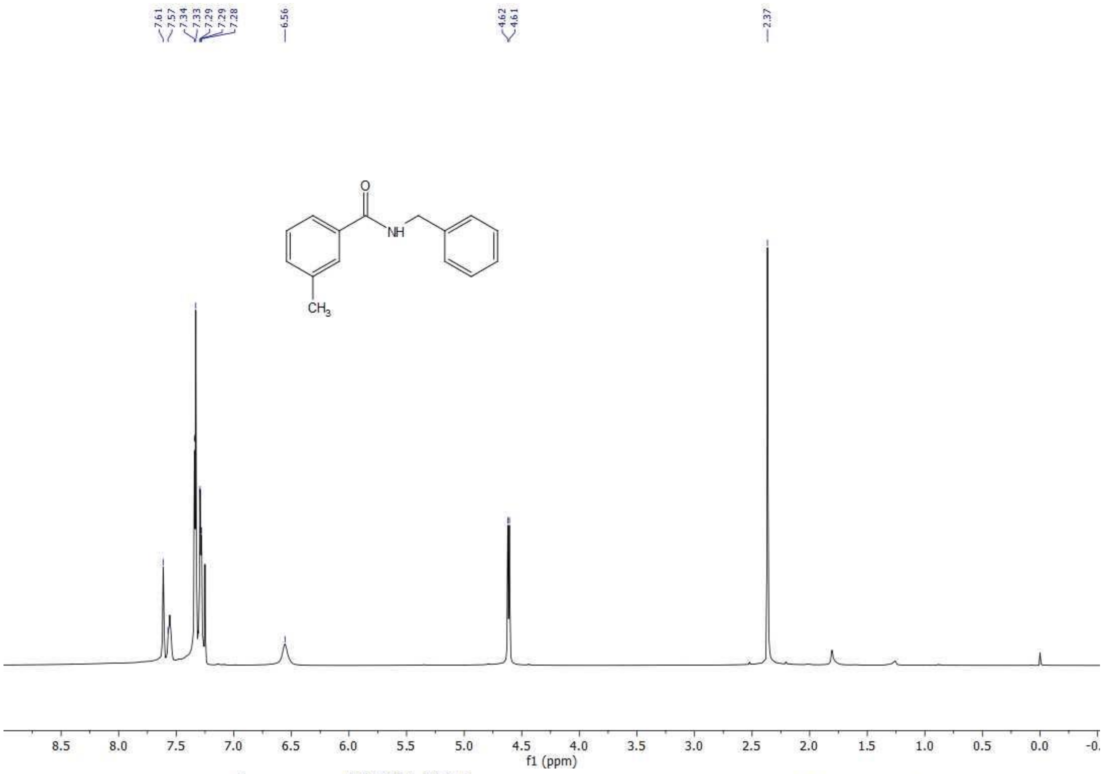
7.61
7.57
7.34
7.33
7.29
7.29
7.28
6.56
4.62
4.61
2.37
O
NH
CH3
8.5 8.0 7.5 7.0 6.5 6.0 5.5 5.0 4.5 4.0 3.5 3.0 2.5 2.0 1.5 1.0 0.5 0.0
f1 (ppm)

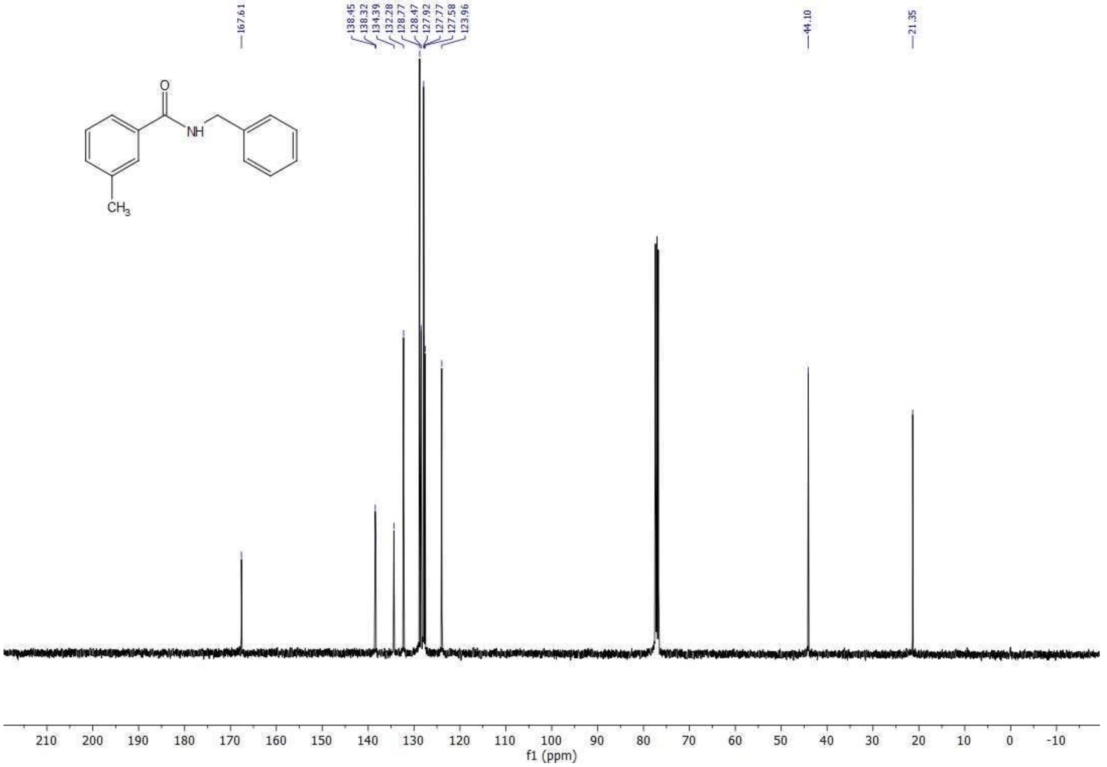
167.61
138.45
138.32
134.39
132.28
128.77
128.47
127.92
127.77
127.58
123.96
44.10
21.35
O
NH
CH3
210 200 190 180 170 160 150 140 130 120 110 100 90 80 70 60 50 40 30 20 10 0 -10
f1 (ppm)

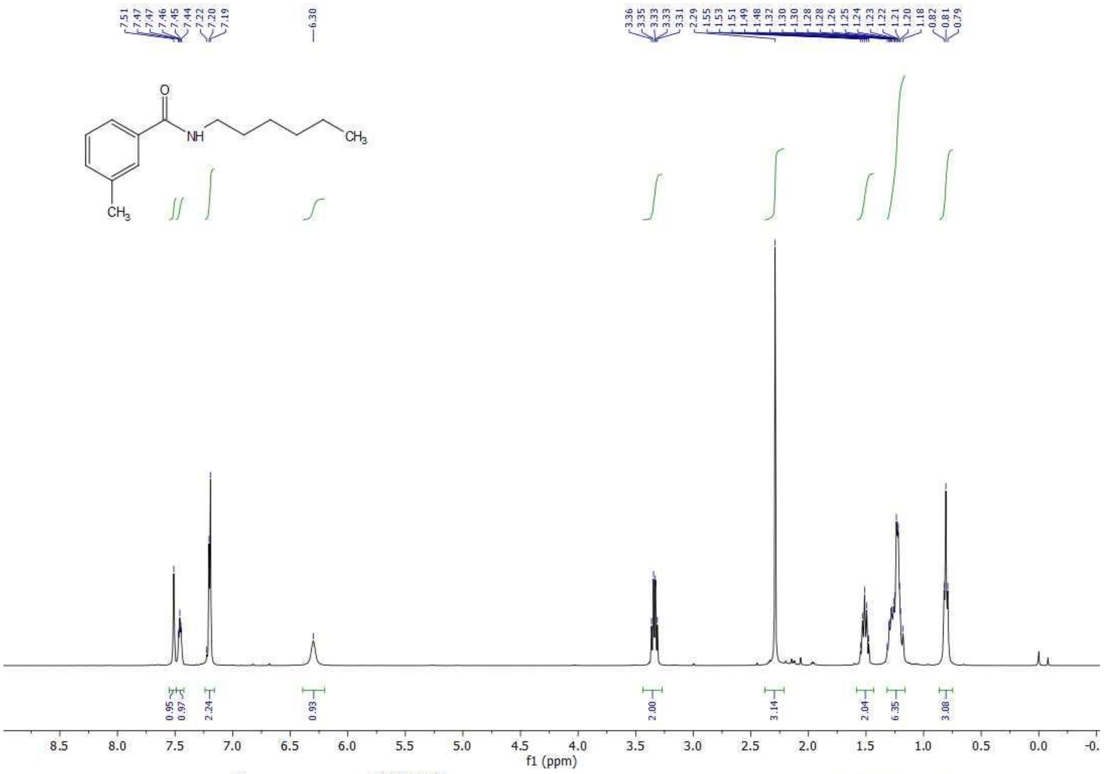
7.51
7.47
7.47
7.46
7.45
7.44
7.22
7.20
7.19
6.30
3.36
3.35
3.33
3.33
3.31
2.29
1.55
1.53
1.51
1.49
1.48
1.32
1.30
1.30
1.28
1.28
1.26
1.25
1.24
1.23
1.22
1.21
1.20
1.18
0.82
0.81
0.79
O
NH
CH3
CH3
0.95
0.97
2.24
0.93
2.00
3.14
2.04
6.35
3.08
f1 (ppm)

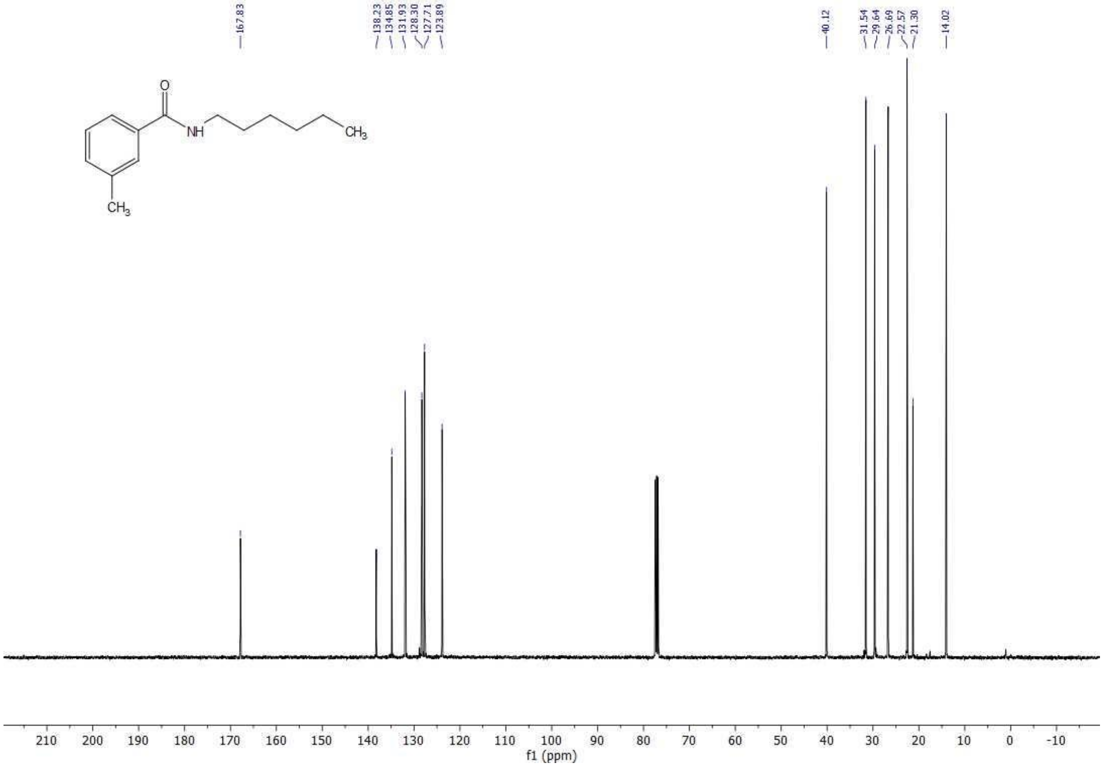
167.83
138.23
134.85
131.93
128.30
127.71
123.89
40.12
31.54
29.64
26.69
22.57
21.30
14.02
O
NH
CH3
CH3
f1 (ppm)

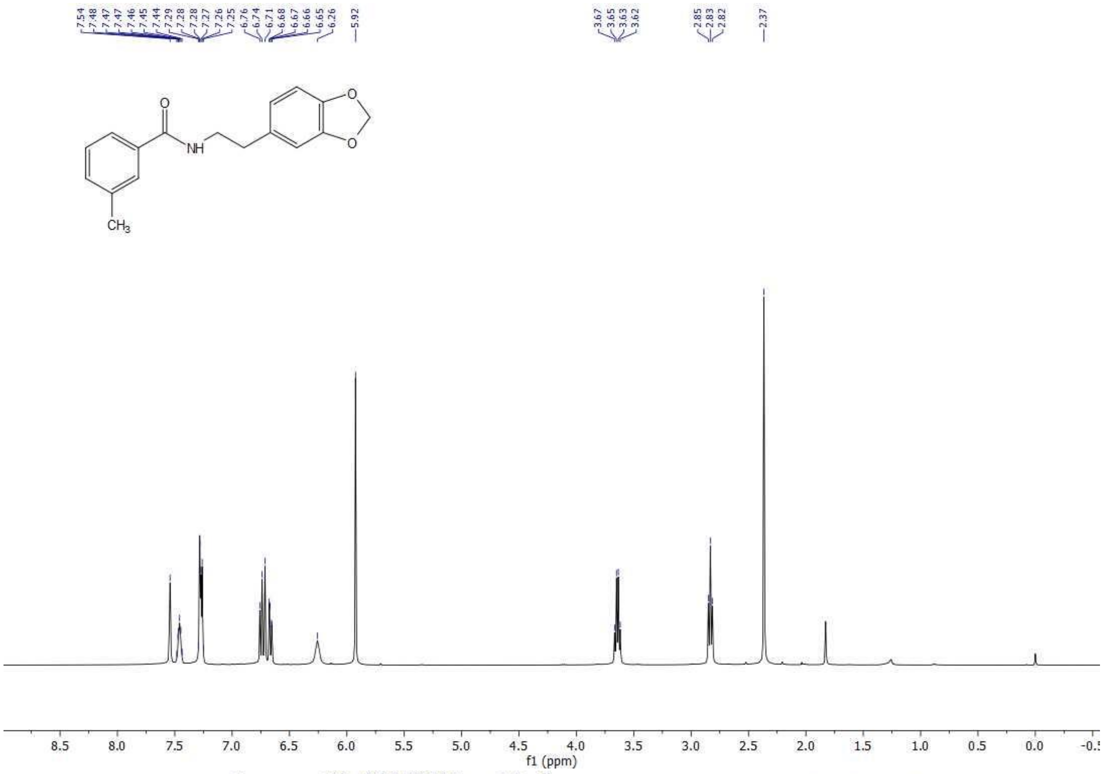
7.54
7.48
7.47
7.46
7.45
7.44
7.29
7.28
7.28
7.27
7.26
7.25
6.76
6.74
6.71
6.68
6.67
6.66
6.65
6.26
5.92
3.67
3.65
3.63
3.62
2.85
2.83
2.82
2.37
O
NH
O
O
CH3
f1 (ppm)

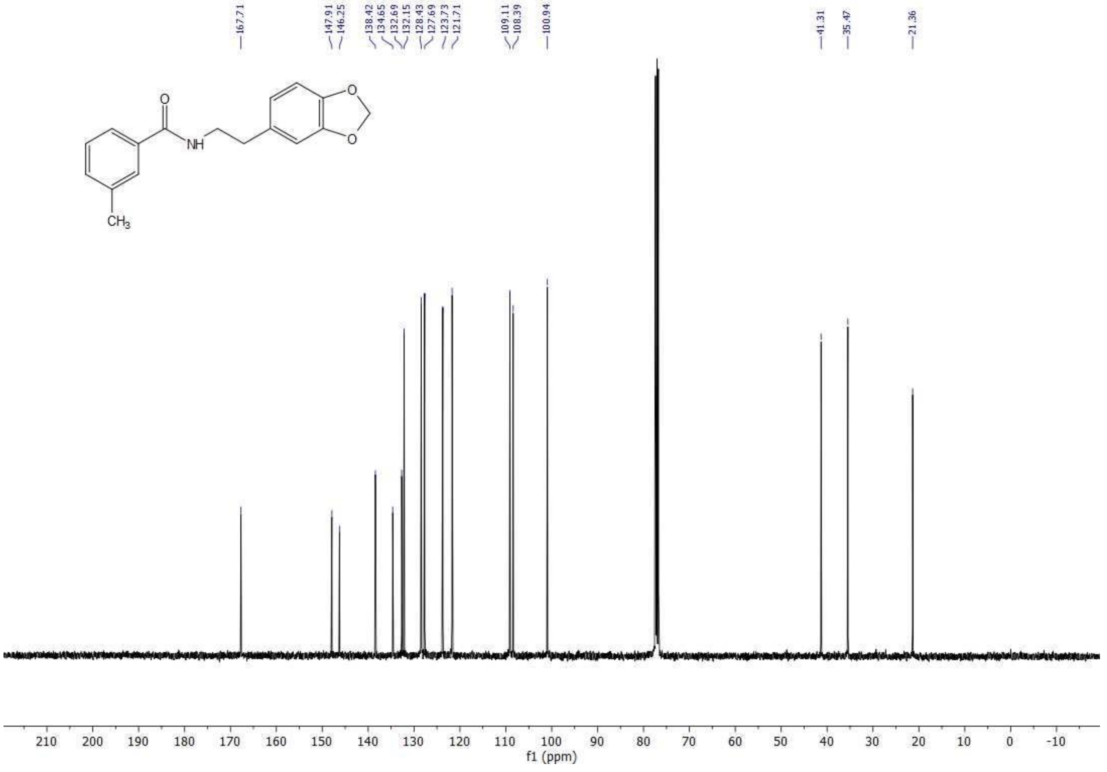
167.71
147.91
146.25
138.42
134.65
132.69
132.15
128.43
127.69
123.73
121.71
109.11
108.39
100.94
41.31
35.47
21.36
O
NH
O
O
CH3
f1 (ppm)

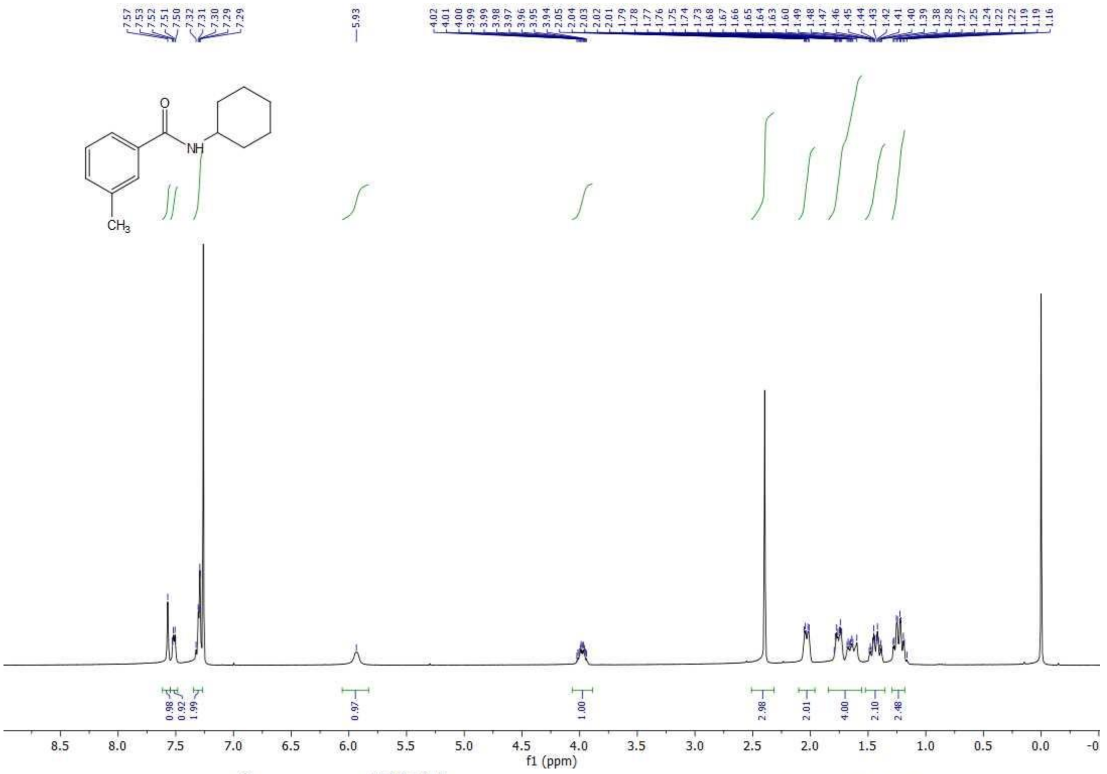
7.57
7.53
7.52
7.51
7.50
7.32
7.31
7.30
7.29
7.29
5.93
4.02
4.01
4.00
3.99
3.99
3.98
3.97
3.96
3.95
3.94
2.05
2.04
2.03
2.02
2.01
1.79
1.78
1.77
1.76
1.75
1.74
1.73
1.68
1.67
1.66
1.65
1.64
1.63
1.60
1.49
1.48
1.47
1.46
1.45
1.44
1.43
1.42
1.41
1.40
1.39
1.38
1.28
1.27
1.25
1.24
1.22
1.19
1.16
O
NH
CH3
0.98
0.92
1.99
0.97
1.00
2.98
2.01
4.00
2.10
2.48
8.5 8.0 7.5 7.0 6.5 6.0 5.5 5.0 4.5 4.0 3.5 3.0 2.5 2.0 1.5 1.0 0.5 0.0 -0
f1 (ppm)

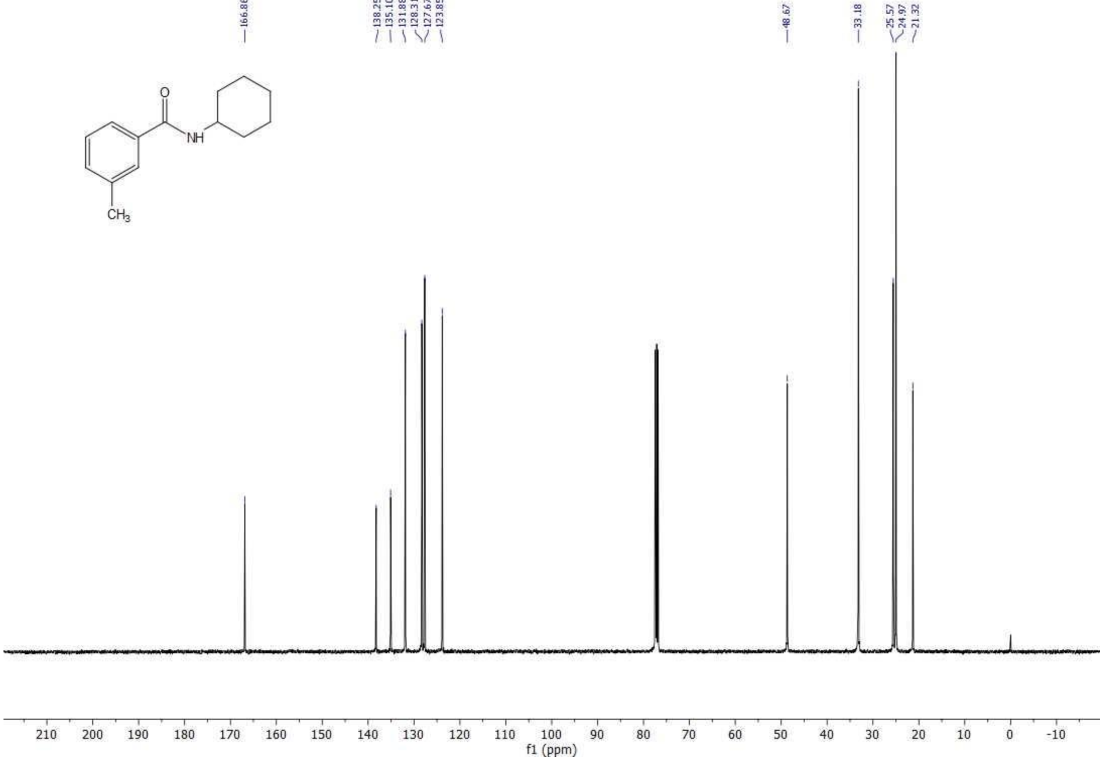
166.86
138.25
135.10
131.88
128.31
127.67
123.85
48.67
33.18
25.57
24.97
21.32
O
NH
CH3
210 200 190 180 170 160 150 140 130 120 110 100 90 80 70 60 50 40 30 20 10 0 -10
f1 (ppm)

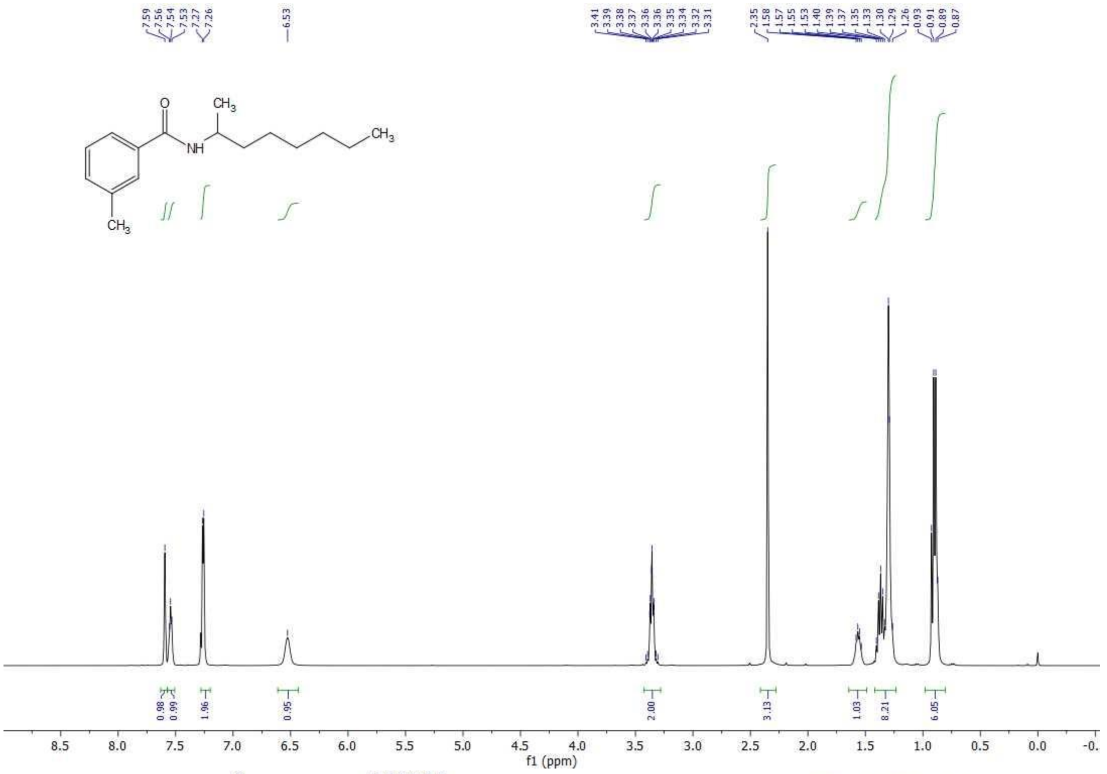

7.59 7.56 7.54 7.53 7.27 7.26
6.53
3.41 3.39 3.38 3.37 3.36 3.36 3.35 3.34 3.32 3.31
2.35 1.58 1.57 1.55 1.53 1.40 1.39 1.37 1.35 1.33 1.30 1.29 1.26 0.93 0.91 0.89 0.87
O
NH
CH3
CH3
CH3
0.98 0.99 1.96 0.95 2.00 3.13 1.03 8.21 6.05
f1 (ppm)

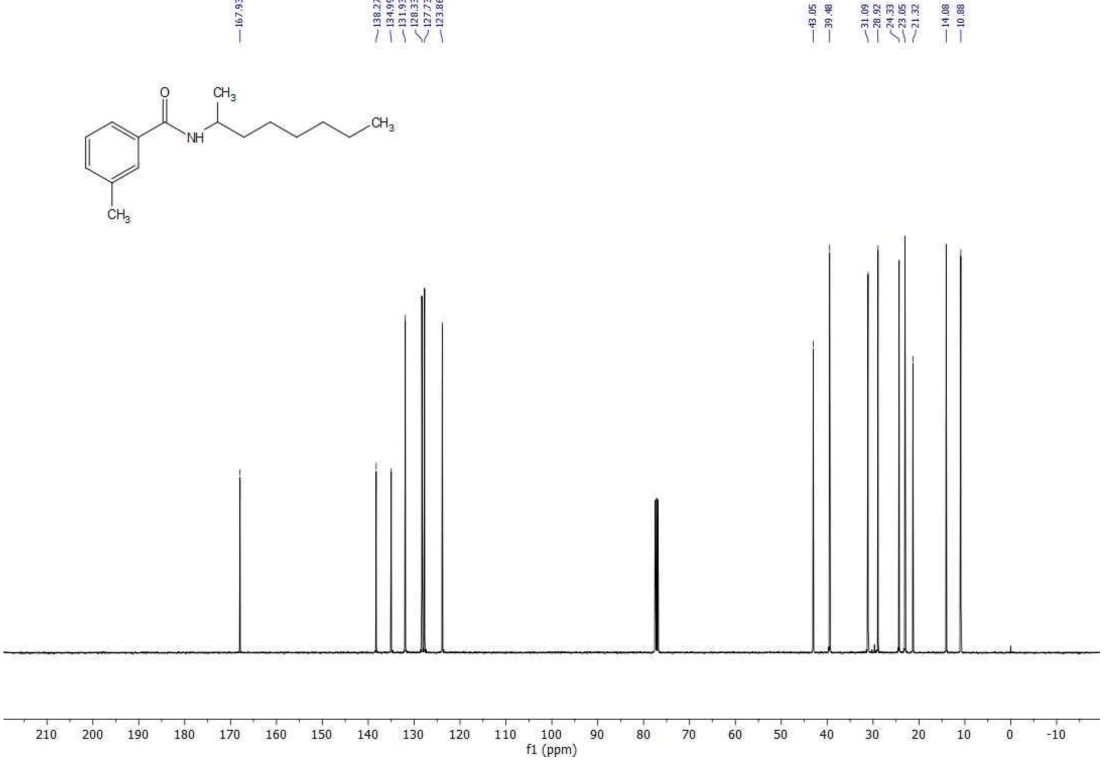

167.93
138.27 134.99 131.93 128.33 127.73 123.86
43.05 39.48 31.09 28.92 24.33 23.05 21.32 14.08 10.88
O
NH
CH3
CH3
CH3
f1 (ppm)

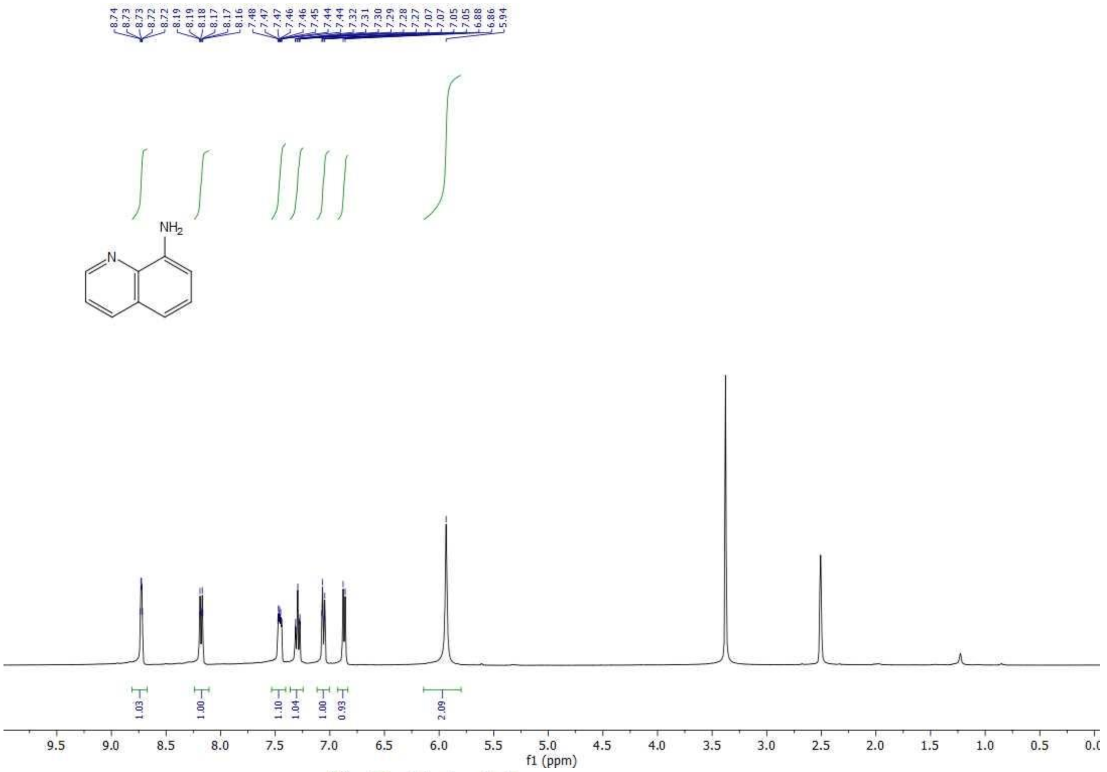
8.74 8.73 8.73 8.72 8.72 8.19 8.19 8.18 8.17 8.17 8.16 7.48 7.47 7.46 7.46 7.45 7.44 7.44 7.32 7.31 7.30 7.29 7.28 7.27 7.07 7.07 7.05 7.05 6.88 6.86 5.94
NH2
N
1.03
1.00
1.10
1.04
1.00
0.93
2.09
f1 (ppm)

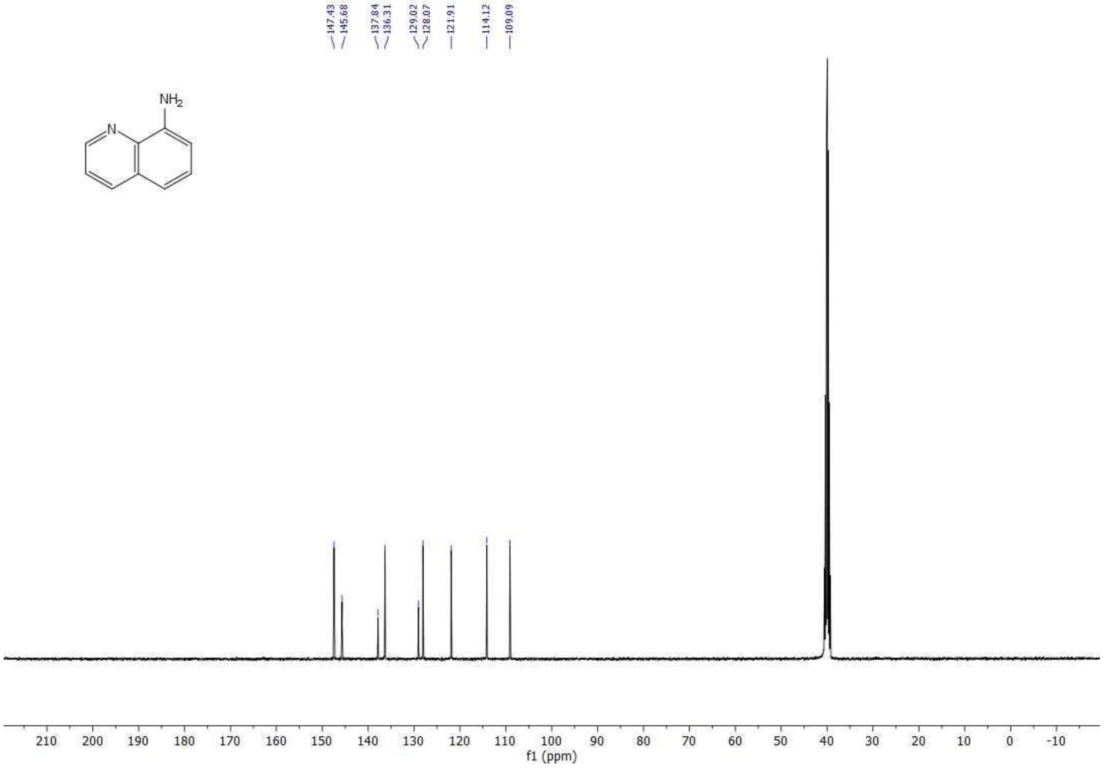
147.43
145.68
137.84
136.31
129.02
128.07
121.91
114.12
109.09
NH2
N
f1 (ppm)
